# Strategies and Tools for Studying Microglial-Mediated Synapse Elimination and Refinement

**DOI:** 10.3389/fimmu.2021.640937

**Published:** 2021-02-23

**Authors:** Raffaella Morini, Matteo Bizzotto, Fabio Perrucci, Fabia Filipello, Michela Matteoli

**Affiliations:** ^1^Laboratory of Pharmacology and Brain Pathology, Neurocenter, Humanitas Clinical and Research Center - IRCCS, Rozzano, Italy; ^2^Department of Biomedical Sciences, Humanitas University, Pieve Emanuele, Italy; ^3^Department of Psychiatry, Washington University School of Medicine, St. Louis, MO, United States; ^4^Consiglio Nazionale Delle Ricerche (CNR), Institute of Neuroscience – URT Humanitas, Rozzano, Italy

**Keywords:** microglia, synaptic pruning, phagocytosis, confocal microscopy, flow cytometry

## Abstract

The role of microglia in controlling synapse homeostasis is becoming increasingly recognized by the scientific community. In particular, the microglia-mediated elimination of supernumerary synapses during development lays the basis for the correct formation of neuronal circuits in adulthood, while the possible reactivation of this process in pathological conditions, such as schizophrenia or Alzheimer's Disease, provides a promising target for future therapeutic strategies. The methodological approaches to investigate microglial synaptic engulfment include different *in vitro* and *in vivo* settings. Basic *in vitro* assays, employing isolated microglia and microbeads, apoptotic membranes, liposomes or synaptosomes allow the quantification of the microglia phagocytic abilities, while co-cultures of microglia and neurons, deriving from either WT or genetically modified mice models, provide a relatively manageable setting to investigate the involvement of specific molecular pathways. Further detailed analysis in mice brain is then mandatory to validate the *in vitro* assays as representative for the *in vivo* situation. The present review aims to dissect the main technical approaches to investigate microglia-mediated phagocytosis of neuronal and synaptic substrates in critical developmental time windows.

## Introduction

Synapse formation is a critical step in the assembly of neuronal circuits. Both secreted and membrane-associated proteins contribute to the formation and maturation of synapses. The process of synaptogenesis is started when initial contacts between synaptic partners are established through filopodia, which lose their motility and become stabilized, to transform into synaptic structures. Synaptic contacts are generated in excess during the early phases of development and therefore, at subsequent stages, the redundant, weak synapses are eliminated, while the more active are strengthened. This selective loss of synapses during a critical period is responsible for structuring neuronal circuits for the remainder of life. In the last years, microglia have emerged as a key player in the process of synapse formation as well as in synapse elimination (1, 2).

Microglia, which derive from myeloid progenitors in the yolk sac, invade the brain around embryonic day 9 in mice (3). As development proceeds, microglia acquire a highly ramified morphology with multiple, motile processes that continuously monitor the brain microenvironment and supervise the neuronal health state. The functional interactions of microglia with neurons are spatially and temporally controlled and comprise several processes including phagocytosis of apoptotic cells, modulation of neurogenesis and regulation of myelin formation (4). Furthermore, microglia have a key role in synapse surveillance, which occurs through the frequent, transient physical interactions between these cells and synapses (5, 6). Short contacts of dendrites by microglia in the somatosensory cortex during the synaptogenesis period were shown to induce filopodia and dendritic spines, via calcium-, actin- and neurotrophin-mediated mechanisms (7, 8), while microglia–spine contacts were associated to the ability of microglia to phagocytose and eliminate synaptic material. To carry out these critical, diverse tasks, microglia assume distinctive states that change over time and which are defined by unique molecular signatures over the course of development (9).

Since the 1970s, neuroscientists have known that synaptic density in the brain changes with age. In 1983, the psychiatrist Irwin Feinberg, at the University of California in San Francisco, described the reduction in spine density as synaptic “pruning” (10, 11). In this process, the removal of weaker structures reallocates resources to those remaining, allowing them to grow stronger and more stable (12, 13). With the clear evidence that synaptic activity guides proper pruning (14, 15), researchers' attention turned to uncovering the cellular mechanisms that might regulate the remodeling. In 2007, Stevens et al. identified an unexpected role for the classical complement cascade in CNS synapses elimination. In particular, they showed that complement proteins opsonize or “tag” synapses in the brain during a discrete window of postnatal development and that the complement proteins C1q and C3 were required for synapse elimination in the developing retinogeniculate pathway (16). These data, combined with the already described phagocytic capacity of myeloid cells, led to the hypothesis that microglia may have a role in phagocytic elimination of synapses as part of the widespread pruning of supernumerary synaptic connections during development.

Consistent with their selective elimination, synaptic components were detected inside microglial phagocytic compartments. However, whether significant portions of synapses are engulfed or small (<1 um) synaptic membrane components are rapidly captured through a process named trogocytosis, is still debated (17). As expected, an excess of immature synapses was detected in mice lacking either the fractalkine receptor Cx3cr1, a chemokine receptor expressed by microglia in the brain (18), or complement components (15). The occurrence of supernumerary synapses was also detected recently in mice genetically lacking TREM2, an innate immune receptor of the immunoglobulin superfamily, expressed by microglia in the central nervous system (CNS), and playing a pivotal role in microglial cell activation, phagocytosis, survival, clustering to amyloid beta (Aβ) plaques [reviewed in (19)]. These recent findings, therefore unveiled TREM2 as a key microglial phagocytic receptor mediating the process of synapse elimination during neurodevelopment (20, 21). The presence of multiple tags seems therefore to be required in order to univocally mark the synapse to be eliminated, while additional protective molecules avoid the inappropriate synapse removal. Among the latter, the “don't eat me” signal CD47 and its receptor, signal regulatory protein α (SIRPα), were found to represent molecular brakes for excessive pruning in the developing retinogeniculate system (22).

In the last years, evidence emerged that the mechanisms of synapse elimination, operating during development, can become aberrantly “reactivated,” and may possibly contribute to pathological synapse loss occurring in neurodegenerative diseases (23). Consistent with this view, both the complement cascade and TREM2 were found as implicated in Alzheimer Disease, with synaptic C1q being aberrantly elevated and contributing to synapse loss (24, 25) and several TREM2 variants being associated to the disease [reviewed in (26)]. Also, a reduction in the synapse-protecting molecule CD47 has been reported in patients with multiple sclerosis (27). Furthermore, several studies described an altered phagocytic function of microglia in Parkinson's Disease [reviewed in (28)]. The occurrence of a concomitant increase of “eat me” signals and decrease of “don't eat me” signals in these diseases, leading to an aberrant microglial phagocytosis and producing synaptic alterations, is becoming therefore a realistic possibility.

Based on these considerations and on the emerging role of abnormal synapse elimination in neurodegenerative processes, we expect that this process will be an increasingly important area of future investigation, also as a potential therapeutic target for reducing excessive phagocytosis in pathological conditions. In this review, we intend to provide a survey of the different technical approaches for studying, both *in vitro* and *in vivo*, the phagocytosis of neuronal and synaptic substrates by microglia. For each of these strategies, strengths and weaknesses will be evidenced, and possible resolution approaches will be proposed.

## Microglia Sources

### Microglial Cell Lines

Despite *in vitro* conditions clearly represent an over-simplified scenario, microglial cultures are doubtless a very useful tool to study phagocytosis, thanks to the possibility to control almost all the experimental settings. Immortalized cell lines are often chosen, due to their ability to proliferate and provide abundant material when the use of animal models is not possible. One of the most frequently adopted cell line are BV2 cells, an immortalized murine cell line obtained by infecting primary microglia with J2 retrovirus carrying v-raf/v-myc oncogene (29). Transformed cells express several macrophage markers, as MAC-1, MAC-2 and IBA1 (30), and are able to develop an adequate response to classical stimuli. For example, LPS stimulates the release of IL1β in BV2 cells (29) and Aβ fibrils promote phagocytosis (31–34). In addition to BV2, the most implemented mouse cell line is N9, which was developed by immortalizing mouse primary microglia with the v-myc or v-mil oncogenes of the avian retrovirus MH2. N9 cells share many phenotypical features with primary microglia cultures. Indeed, N9 cells express the microglial markers FcR, Mac-1, and F4/80 (35) and two purinergic receptor subtypes, metabotropic (P2Y) and ionotropic (P2Z) (36). As for primary microglial cultures, they respond to TNFα stimulation with a reduction of the expression of the SR-A and CD36 and also in Aβ uptake (37). Moreover, LPS stimulation induces the release of IL-6, TNFα, and IL-1β in N9 cell line (35). Further additional cell lines include the colony stimulating factor-1 dependent EOC cells (38), C8-B4 and RA2 cell line which are not genetically modified (39–42). Although these cells have been widely adopted in several studies, related in particular to inflammation (43), it is increasingly clear that data obtained from cell lines need to be compared to results from primary microglia and *in vivo* models, to be considered as reliable (44). Indeed, prolonged culturing of cell lines can negatively influence their characteristics. After many generations, immortalized cell lines can suffer of duplications or chromosomes rearrangement, therefore, mutations and epigenetic changes risk to accumulate over time (45, 46). Das et al. adopted an RNA-seq approach to finely distinguish the differences in gene expression between primary cultured microglia cells and BV2 after LPS treatment (47). Primary microglia had a stronger response to the stimulus and the expression of numerous cytokines, chemokines and interferon regulated genes was uniquely affected, for example IL12 and CCL5, whose increased levels have been associated to neuroinflammation [(48, 49) for a more detailed list, see (47)]. A few years later, Butovsky et al., showed that the microglial cell lines N9 and BV2 do not express any of the genes characteristic of the TGF-β–dependent adult microglia signature (50).

### Primary Newborn Microglia

**Figure 1:**
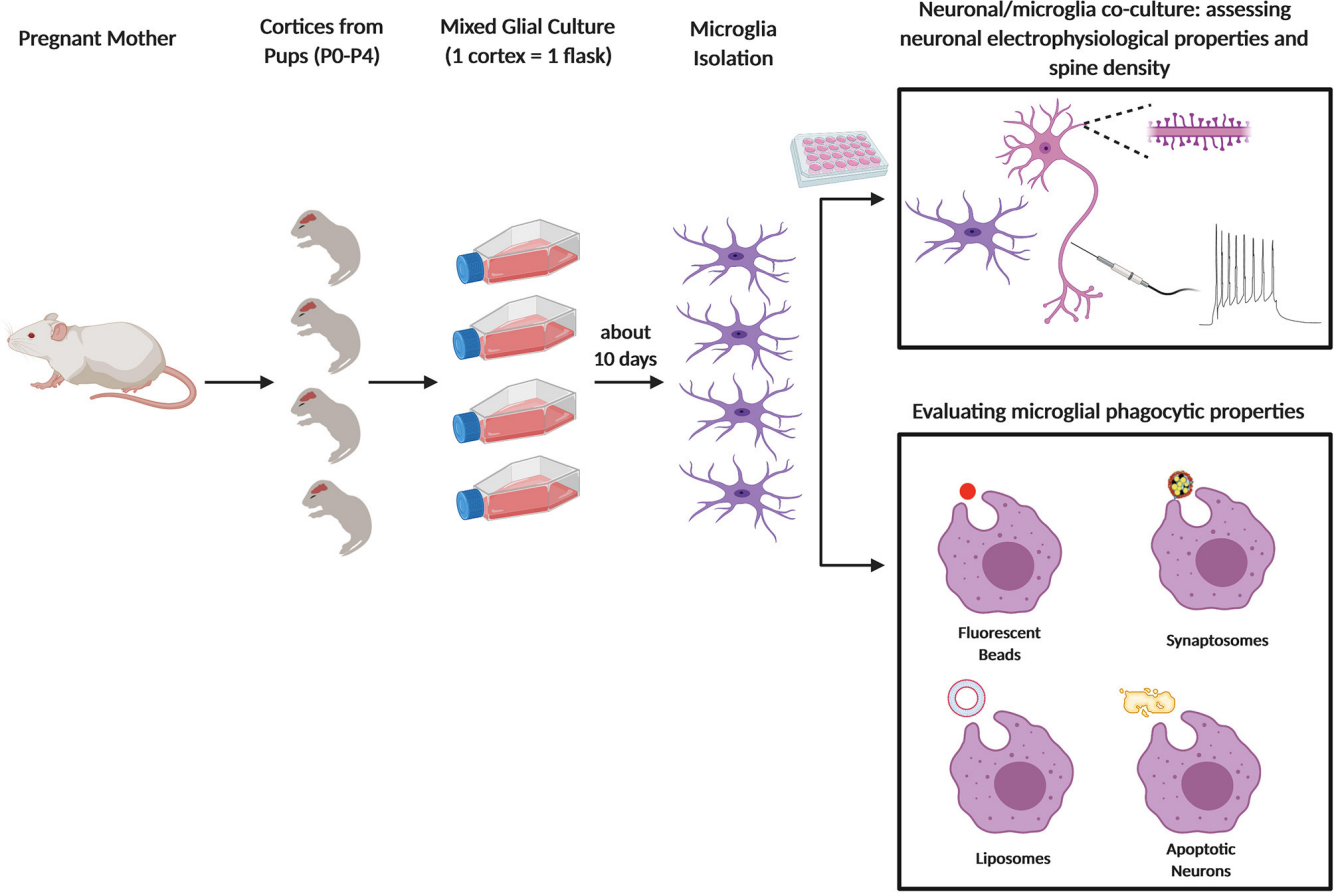
Primary microglia culture and main usages to assess the phagocytic process. Schematic figure depicting newborn mice (P0–P4) from pregnant female used to obtain primary microglia culture. After dissection and enzymatic digestion of cortices and hippocampi, cells are resuspended in growth medium (usually composed by either DMEM or EMEM with 10 to 20% of FBS), to sustain microglial growth. Cells are plated in T75 flasks and cultured for at least 10 days at 37°C. Microglia are subsequently collected either by vigorously tapping the flasks, by agitation at 230–245 rpm for 45 min or through mild trypsinization. In some assays, microglia are cultured alone or co-cultured with neurons and the cells are analyzed by fluorescent microscopy (e.g. to quantify neuronal spine number) or by electrophysiology (top panel); in other assays, microglia phagocytic properties are assessed by feeding the cells with specific substrates: fluorescent beads, synaptosomes, liposomes, or apoptotic neurons (bottom panel).

Relative to cell lines, more advisable is the use of primary microglia, that can be isolated from embryos and newborn mouse pups in the P0–P4 time window (51, 52) (Figure 1). Dissociated cells are collected through enzymatic digestion of mouse brains and seeded as mixed glial culture. Microglia growing on top of a confluent astrocyte layer, generally in 2 weeks, are next purified through mechanical tapping of mixed glial culture [for a protocol see (53)]. After 2 h, microglia attach to the bottom and, after replacement with fresh culture medium, are ready to be used, starting from the next day. The use of primary microglia allows to perform *in vitro* assays in controlled conditions, with a relatively short time interval from the cultured cell collection to their employment (39). Although representing an advancement toward the use of immortalized cell lines, the use of cultured primary microglia suffers of important limitations. First, local environment is known to exert a profound influence on microglia, and indeed it is widely recognized that microglia quickly lose their transcriptional phenotype after niche removal (54, 55). In addition, the use of media containing serum, which are usually adopted to ensure vitality and proliferation of freshly isolated microglia, results in a low reproducibility of data, due to batch-to-batch heterogeneity (39). Since factors required for microglia survival can be found in media conditioned by astrocytes (56), a number of protocols for culturing primary microglia from newborn mice use mixed cultures composed by a confluent layer of astrocytes on which microglia grow in semi-suspension (21, 53). Although listing the different methods for isolating and culturing primary microglia is not the purpose of this review, possible hints to at least partially overcome these issues are discussed in the relative chapter.

### Primary Adult Microglia

**Figure 2:**
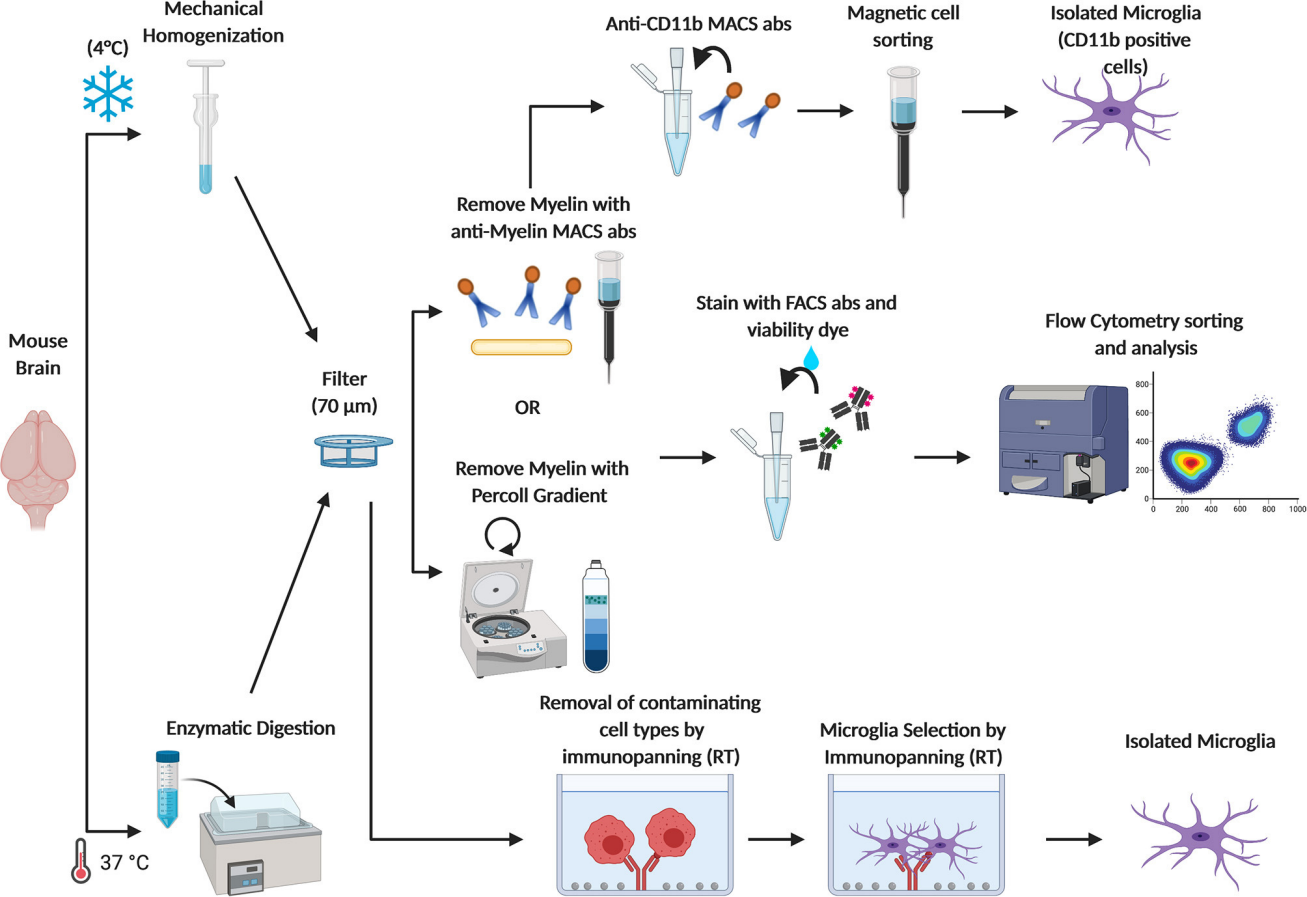
Schematic figure depicting current methods adopted to obtain freshly isolated microglia. Brains from adult mice are (1) homogenized with a dounce homogenizer at 4°C to avoid microglia activation or (2) enzymatically digested at 37°C in the water bath, and filtered through a 70 μm cell strainer. Myelin is removed either by centrifugation with a Percoll gradient or by using anti-myelin magnetic cell sorting (MACS) antibodies. Then, microglia are sorted from other CNS cell types by FACS through specific cell markers. Otherwise, microglia are isolated by MACS anti-CD11b antibodies. Microglia can also be isolated by immunopanning. In this approach, the cells suspension is passed over a series of antibody coated dishes in order to remove contaminating cell types. Then, microglia are positively isolated in the last coated dish.

Because of the clear evidence of the central role played by microglia during physiological and in pathological context, the possibility to isolate intact microglia from the adult brain has become very appealing through the years and has been pursued by many groups. Microglia isolation from the adult brain presents some challenges, and several protocols have been published and optimized along the way (Figure 2). One of the first studies describing a successful method for isolating microglia from human and rat brain homogenates, was carried out by M. L. Cuzner's group in 1988 (57), followed by another work from Volker Ter Meulen's group a few years later (58). These protocols are based on an initial enzymatic digestion followed by separation steps using a Percoll gradient of various densities that allows separating myelin debris from nervous cells. Over the years, this procedure has been improved and optimized. Indeed, while until 2015, homogenization of the whole brain or of specific brain areas was mostly performed by enzymatic digestion (by using enzyme like Collagenase D, Dispase, Trypsin, and or Papain) carried at 37° or at room temperature (RT) (51, 59–66), more recently Ben A. Barres' group modified the existing microglia isolation protocols in order to minimize microglia activation during the isolation procedure. The whole procedure is now carried out under consistently cold conditions (on ice or at 4°C) and the brains are mechanically homogenized using a dounce homogenizer instead of undergoing to enzymatic digestion. Flow cytometry and RNAseq expression of cell-type–specific markers showed that avoiding enzymes and maintaining cold temperatures throughout the whole isolation process prevented transcriptional phenotypic changes and hyper activation of isolated microglia (9, 67). Furthermore, a reliable cell separation is now successfully obtained through the following three approaches: (1) Fluorescence activated cell sorting (FACS), (2) Magnetic-activated cell sorting (MACS), and (3) Immunopanning (Figure 2).

#### Fluorescence Activated Cell Sorting (FACS)

This is the most widely used approach where microglia are sorted with a high cell purity from other major CNS cell types through immune cell markers. CD45 and CD11b, which are not present on the surface of other glial cells or neurons, are commonly used to identify microglia (20, 59, 60, 68–70). Microglia are CD45^low^CD11b^+^ and can be therefore distinguished from monocyte and macrophage populations (CD45^high^CD11b^+^) (58). However, since the separation based on CD45 expression levels is not sufficient to cleanly separate microglia from all the other myeloid populations, such as neutrophils or choroid plexus macrophages, many groups recently invested increasing efforts in order to identify unique and highly specific markers to selectively distinguish microglia. The Transmembrane Protein 119 (tmem119) and the Purinergic Receptor P2y12 have been shown to be exclusively expressed by microglia and have been added to the sorting procedure (50, 67, 71). More recently, the hexosaminidase subunit beta (Hexb) has been described as a stably expressed microglia core gene, with a rather stable expression also during inflammation and neurodegeneration (72).

#### Magnetic-Activated Cell Sorting (MACS)

This approach is based on the use of anti-CD11b immunomagnetic beads. The anti-CD11b antibodies recognize CD11b surface antigens on microglia by positive selection. Since these antibodies are conjugated to magnetic beads, they allow the retention of labeled cells in a magnetic field. Therefore, this strategy efficiently selects CD11b^+^ cells over other major CNS cell types, and the large majority of CD11b^+^ cells from the uninjured CNS are in fact microglia (63, 73). Myelin debris can also be removed using the same immunomagnetic beads approach, instead of using Percoll gradient.

#### Immunopanning

In this strategy, antibodies recognizing CD11b surface antigens are immobilized on a Petri dish and used to retain microglia from brain single-cell suspensions. Panning is trivial, involving only three steps: (1) enzymatic preparation of a cell suspension, (2) passing this suspension over a series of antibody-coated dishes, and (3) removing the purified cells from the final dish. This protocol has been less commonly used (74–76).

After isolation, fresh microglia from adult mice can be cultured *in vitro* or directly assayed for their functional and phagocytic properties. Many protocols have been developed to maintain adult microglia in culture for several days. Some groups showed the generation of pure microglia from adult mice and their maintenance in culture for more than 60 days starting from a mixed glial population treated with GM-CSF (77). Yet, these cultures maintain a high proliferative capacity, which might be due to an immature phenotype of these cells, since adult microglia is not mitotically active nor proliferate in response to GM-CSF or M-CSF (50, 59, 78).

More recently, Bohlen et al., shed a clearer light on previous procedures and proposed a new method to maintain adult microglia in culture. They were able to successfully culture microglia from juvenile or adult rat brains, but they observed that microglia cultures from old mice (>P14) were not viable. Cultures from mice younger than <P14 were viable, although the yields and survival rates were lower if compared to rat tissue. So, the authors concluded that microglia cultures from rat and mice should be performed starting from young animals since cell yield and viability drop with increasing animal age. Once isolated, microglia were maintained in culture in the presence of TGF-β2 and IL-34 or M-CSF of absence of FBS (75). Due to the challenges of maintaining adult microglia *in vitro* and after the discovery that microglia lose many of the core signature genes such as Tmem119 and P2ry12 only after a few days *in vitro* (50, 56, 79), a limited number of studies have performed phagocytic assays on adult microglia cultures. Indeed, most of the phagocytic assays currently described in literature are performed using microglia prepared from newborn mice as we described in the first section. This is still a reliable and useful system, in which the cells are easier to obtain and can be cultured for a longer period of time (20, 21).

### Human Microglia-Like Cells

Recently, different groups demonstrated the existence of significant differences between murine and human microglia (79–81). This further highlighted the importance of finding new models to better understand the genetics and function of human microglia. With this purpose, a large effort has been done by the scientific community to generate human microglia-like cells (iMGLs) from human embryonic stem cells (ESCs) or by the reprogramming of adult cells (i.e., fibroblast or blood cells) into induced pluripotent stem cells (iPSCs) via the overexpression of specific transcription factors.

Several detailed methods for the generation of iMGLs have been published in the past 4–5 years (82–87). The common thread of these new generation protocols is that the specific steps through which iPSCs are differentiated into microglia-like cells, seek to mimic microglia ontogeny.

Indeed, developmental ontogeny studies showed that microglia are of mesodermal origin, deriving from erythromyeloid progenitor (EMP), that arise from the yolk-sac (3, 88, 89). Therefore, the new methods generate cells that transition from iPSCs to primitive hematopoietic precursor cells (HPCs), EMPs, and, ultimately, microglia.

iMGLs phenotype has been shown to be induced by incubation of human iPSC-derived microglial and/or macrophage progenitors with various combinations of cytokines, including high levels of CSF-1 and IL-34 (85); IL-34 and GM-CSF (83); and IL-3, IL-34 and GM- CSF (86). In order to recreate the brain environment, and to push iMGLs maturation further, some of these protocols also proposed to co-culture iMGLs with neurons (84, 87) or to add further cytokines such as TGF-β1, CX3CL1 (also known as fractalkine), and CD200 which are critical for microglia homeostasis and to mimic neuronal proximity (82) [reviewed in (90)].

Moreover, microglia-like cells from iPSCs allows the comparison between healthy donors and patients with neurological disorders. This aspect is of primary interest and, together with further recent improvements, such as the addition of iMGLs to iPSC-derived brain organoids or the xenotransplantation of HPCs/ iMGLs into mouse brain, makes iMGLs a powerful system to study properties and dysfunctions of human microglia [reviewed in (91)].

### Pitfalls and Hints

Although primary microglia isolated from embryonic (92) or neonatal mice and rats are widely used as *in vitro* models, recently it has become evident that the tissue environment has a major impact on microglia transcriptome (50, 56, 79). Despite the important advances that have been made to improve culture conditions of microglia and iPSCs-derived microglial cells (39), *in vitro* microglia, although being informative and providing a useful setting to dissect basic mechanisms and possible dysfunctions of phagocytic microglia (20, 21, 50), is far from recapitulating the profile and function of microglia in their physiological environment.

As mentioned, the main limit of microglial culturing is the wide adoption of medium containing serum for their maintenance. Fetal bovine serum is usually added to medium to a final concentration of 10–20% in order to promote microglia proliferation and survival (20, 21, 53). However, microglia are not exposed to serum proteins in the brain and FBS perturbs microglia phenotype *in vitro* (39, 56), thus increasing the risks of *in vitro* artefacts. A solution to this problem has been provided by Bohlen et al., who identified in CSF-1, TGF-β and cholesterol the minimum supplement requirement for microglia culturing, a condition which allows obtaining an *in vitro* model with a significantly higher reproducibility (56).

The authors purified microglial cells from postnatal rat brain by immunopanning, and quantified their viability 5 days after plating. As expected, microglia showed a high mortality when serum was removed from the medium, but a robust pro-survival rate was reached by culturing microglial cell in a medium preconditioned by astrocytes. As CSF-1 and TGF-β were not sufficient to promote microglia viability, they cleverly dissected the conditioned medium and added cholesterol as the third key element, obtaining the so-called TIC medium (TGF-β2 2 ng/mL, IL-34 100 ng/mL, and cholesterol 1.5 mg/mL) (56). More recently, the Seker's group established an innovative tri-culture of neurons, astrocytes and microglia adopting the same cocktail used in TIC medium. Under these conditions, microglia showed a neuroprotective role when neurons were exposed to excitotoxic events, and the response to external stimuli mimicked neuroinflammatory responses better than classical co-cultures (93). Nevertheless, in contrast with Bohlen's results, cytokines detected in unstimulated TIC medium reflected slightly inflamed state and microglia exhibited an amoeboid morphology. As suggested by the authors, it should be considered that the two systems differ in the age of mice from which cultures were obtained. Moreover, the tri-culture medium includes also B27 supplement, whose elements could influence microglial cell phenotype (56, 93).

Another critical issue in microglia culturing resides in the process of cells collection after *in vitro* maintenance. Microglia proliferate in semi-suspension, above a layer of astrocyte and shaking of the culture support (flask or petri-dish) for a defined time and speed is sufficient to detach microglia from the astrocytes layer, re-suspending them in the medium (20, 21, 53). After 10–15 days, the shaken culture will be re-populated by new microglia that could in principle be employed for a new cell collection, the so-called “second-shaking.” A main pitfall in this process is the clonal-selection of a sub-population from the original culture, that makes it only partially comparable to a fresh culture (94). Moreover, the shaking process can stress the cells, inducing phenotypic variations in primary microglia. For these reasons, a mild trypsinization protocol has been adopted as an alternative. Lin et al., compared shaking vs. mild trypsinization (95), demonstrating that microglial morphology and cytokine expression vary depending on the methodology of isolation. Indeed, the shaking protocol induced a higher expression of microglia activation markers, iNOS, CD86, CD206, and arginase 1, together with pro-inflammatory cytokines, TNFα, IL-1β, IL-10, and IGF-1 (95), although both conditions fully maintained microglia ability to respond to classic stimuli, such as IL-4, LPS, and IFNγ (95). Interestingly, by analyzing a panel of genes commonly upregulated during aging or after LPS or Aβ stimulation, it was found that CD11b magnetic-associated cell sorting (MACS) guarantees the highest expression of *Tgfbr1* and *Tgfbr2* genes, and results in a “more quiescent” microglia phenotype, as compared to cells obtained by mild trypsinization and shaking (96). Thus, cell manipulation can heavily influence the microglia condition. Caution should therefore be applied when performing experiments and well-defined control conditions are mandatory to be applied.

Moreover, as already mentioned, the microenvironment exerts a strong effect on the microglia transcriptome (50, 56, 79). In particular, human and mouse microglia *in vitro* cultures exhibit down-regulation of genes characteristic of the core transcriptome signature of microglia and, on the other side, upregulate genes typically only observed *in vivo* in the context of disease or injury (56, 70, 79).

Microglia isolation from the adult brain also presents several challenges. This is primarily due to the fact that microglia are highly responsive to CNS tissue damage, which is inevitable during their isolation, and easily undergo hyper-activation and gene transcription changes after manipulation (9, 67). Another reason is that the final yield obtained after isolation is very low since microglia only account for 5–12% of the total cells in the brain (97–99) [the total yield per brain expected after isolation ranges between 5 and 10 × 10^4^ cell from mice between postnatal (P) days 10 to P21] (75).

Furthermore, the procedures used to isolate and select microglia from adult brain have some disadvantages that need to be considered, especially depending on the use to be done with microglia after their sorting. FACS, the first-choice procedure, allows to obtain a very high cell purity and it is widely and successfully used. Yet, it requires specific and very well-organized FACS facilities, instrumentations that need to be always up to date, and specialized technicians able to manage and use sorters optimally. Another caveat of cell sorting is that the detection antibodies remain bound to the cells at the end of the process, blocking the epitopes and potentially impacting cell function. Also, sorting procedures cause hydrodynamic stress to the cells, even though it has been demonstrated that this does not affect cell structure or function. The second method, based on positive selection of CD11b^+^ cells through magnetic beads, is in fact highly effective but requires significant upfront investment in reagents and equipment that are particularly expensive. A disadvantage of this approach is that positive selection utilizes cell receptor antibodies to target the specific cell type of interest and may potentially turn on activation cascades through these receptors or cause receptor blockade and inhibit the downstream functions of the isolated cells. Moreover, these protocols do not separate microglia from barrier-associated macrophages, monocytes, neutrophils, or certain B cells also present in the tissue. The third strategy, immunopanning, requires minimal reagent investment or specialized equipment but does not provide a high specificity. Indeed, separation of different myeloid populations is unlikely to be achievable using immunopanning due to the propensity of various myeloid cell populations to adhere to the panning dish, even dishes not coated with antibodies. Moreover, this protocol requires cells trypsinization but on the other side, avoids introduction of magnetic particles in downstream applications. Therefore, this approach is not preferable if the final goal is to isolate a pure and homogeneous microglia population.

## *In vitro* and *ex vivo* Engulfment Assays

Once microglia are isolated from brain and deposited in culture, their phagocytic properties can be evaluated using several different substrates (Figure 1). Below we describe some of the assays which can be used to test microglia phagocytosis. Although the focus of this review is on microglial synapse elimination, in this chapter the strategies and the tools that can be used to analyze the basic phagocytic activity by microglia will be described (see Table 1). These assays may provide suitable control conditions, needed to complement the study of synapse elimination by microglia. It is to be considered that the receptors and molecular machineries that coordinate phagocytosis and digestion are likely to differ depending on the specific substrate. Importantly, the substrates and phagocytic events described in this section can only partially model the process of synapses and neurites phagocytosis *in vivo*.

**Table 1:** *In vitro* engulfment assays.

**Samples**	**Type of cells**	**Engulfed substrates**	**Techniques adopted**	**References**
Cell lines	Microglia cell line BV-2	Fluorescent beads	Fluorescent microscopy, Flow cytometry	(100)
	Microglial cell line MMGT12	Fluorescent beads	Flow cytometry	(101)
	Microglial cell line BV-2	Synaptosomes	Fluorescent microscopy	(102)
iPC-derived microglia	Induced Microglia Like Cells (iMGL)	Synaptosomes	Fluorescent microscopy	(103, 104)
Primary cultures	Newborn microglia	Fluorescent beads and liposomes (DiO Labeled)	Fluorescent microscopy	(21)
		fluorescent beads	Fluorescent microscopy, Flow cytometry	(20)
		Fluorescent bioparticles	Fluorescent microscopy	(105)
	Adult microglia	Ultraviolet-irradiated (UV-irr) dead neurons	Fluorescent microscopy	(78)
		Fluorescent microspheres	Fluorescent microscopy	(62)
	Macrophages	Bacteria and cancer cells	CyTOF	(106)

*The table reports some commonly used experimental approaches to study microglial phagocytosis in vitro*.

### Fluorescent Beads

Latex beads have been widely used to analyze the basic phagocytic process by microglia. This type of assay is advantageously used to demonstrate that the phagocytic machinery of microglia is properly functional, even when synapse elimination may be defective. Further, purification of phagosomes containing engulfed latex beads has allowed to understand the phagosome biology on a biochemical and functional standpoint and to dissect the sequential steps at the basis of this process (107–109).

The use of Fluorescent Latex Beads (FLB) has allowed a fast and quantitative analysis of phagocytosis in different cell populations either by FACS (101, 110, 111) or by a simple count of FLB internalization by fluorescence or confocal microscopy (112, 113). FLB, which are routinely used to calibrate flow cytometers, may be excited by a specific wavelength or, alternatively, contain a mixture of fluorophores that enable them to be excited at any wavelength of UV and visible light. FLB have a wide range of sizes (the most commonly used range from 0.5 to 6.0 μm) and are inert, so they are not toxic and do not interfere with cell viability (100, 101). FLB may be either used without any modifications (20, 21, 100, 114, 115) or pre-opsonized with FBS or BSA to improve phagocytosis by microglia (101, 116), since it has been shown that the engulfment of synapses is strictly dependent on complement proteins deposition, such as C1q and C3, and their interaction with microglial cells (24, 103). Moreover, beads can be also carboxylated so to add a negative charge, a model that can be used to mimic negative surface charge of phosphatidylserine-exposing cells (117). The engulfment rate is dependent on FLB concentration and incubation time (115). To precisely evaluate microglia phagocytic capacity, FLB amount and time of incubation need to be precisely set.

Pathogen-associated molecular patterns (PAMPs), such as LPS, significantly increase FLB internalization by microglia (101, 115). Also, in line with the observation that FLB internalization by microglia is accompanied by an increase in TNF-α and TGF-β production (69), MDG548, a neuroprotective PPARγ agonist used for experimental Parkinson's Disease treatment was found to increase FLB engulfment, while decreasing TNFα levels, thus providing a possible basis for PPARγ agonists protective role (101).

Besides PAMPs, basic phagocytic activity by microglia is also enhanced by neurodegeneration-associated molecular patterns (NAMPs), which include the Aβ and neurofibrillary tangles (118, 119). This activation modifies microglial phenotype, turning them into disease associated microglia (DAM) (120). In this framework, Nagano et al., showed, by both confocal microscopy and flow cytometry analysis, that the presence of Aβ deposits is able to increase the engulfment rate of FLB in primary rat microglia. This effect is reversed after the treatment with Prostaglandin E2 (PGE2), through the involvement of microglial E-prostanoid receptor 2 (EP2) (121). Allendorf et al., confirmed that treatment of primary rat microglia with pro-inflammatory stimuli such as LPS, Aβ or Tau induces an increase of FLB phagocytic activity (100). Finally, Yin et al. (111) have shown that the inhibition of EZH2, the catalytic subunit core PRC2, a gene involved in silencing a number of tumor suppressor genes, is able to increase the levels of pro-inflammatory cytokines and the FLB phagocytic capacity of microglia, which are abundant in the tumor environment (111, 122).

Of note, the engulfment of FLB can be also assessed *in vivo*. Hughes et al. (114) injected FLB [6 μm] intrahippocampally in ME7 mice, a model of prion disease, in order to study their engulfment capacity. They discovered that microglia in the degenerating brain, internalize FLB and apoptotic cells, demonstrating that the phagocytic machinery of the microglia in ME7 mice is properly functional.

### Liposomes

An additional substrate that can be successfully exploited for phagocytosis assays are liposomes, or unilamellar vesicles (UVs) (123), a useful tool to specifically investigate the nature of “eat-me” signals which need to be exposed by the target membrane to allow microglial phagocytosis (21).

UVs can be distinguished in three categories depending on their size: small UVs (SUV), large UVs (LUV) and giant UVs (GUV), having a diameter of 20–100, 100–1,000 nm, and 1–200 μm, respectively. UVs stability depends on the experimental conditions; indeed, oxygen reactive species react with unsaturated fatty acid chains, thus altering lipid properties and liposomes structures (124). This responsiveness to the environmental conditions has been exploited for improving drug delivery, through the generation of smart vesicles able to deliver drugs to the target and release them only after a local stimulus-response (124). One of the biggest advantages of this tool is that UVs with virtually any composition can be prepared, enriching them with specific membrane proteins or different lipids (125, 126). For example, in a recent paper, a convenient protocol was published for the preparation of proteo-GUVs containing functionally active neuronal SNARE (soluble N-ethylmaleimide-sensitive factor activating protein receptor) proteins for the study of membrane fusion *in vitro* (125). More recently, the Matteoli's group took advantage of DiO labeled liposomes composed of variable amounts of phosphatidylserine (PS) and cardiolipin (CL) to investigate whether exposed PS impacts microglia ability to engulf lipidic membranes (21). Specifically, the researchers incubated for 1 h liposomes endowed with different lipidic composition with microglial cells isolated from mice either WT or genetically lacking TREM2, a receptor which shows high affinity for phospholipids as phosphatidylcholine and PS (127). Confocal analysis of liposome engulfment inside CD68-positive phagolysosomal organelles in Iba1-positive microglia (15, 21) exploiting the Bitplane Imaris software to generate a 3D reconstruction of the fluorescent signal, allowed to demonstrate that the extent of PS positively correlates with microglia phagocytosis (127). For this kind of experiments, attention should be paid to the type of solvents used for permeabilization before the staining (128). Specifically, the use of saponin allowed to selectively create pores the cholesterol shaft of the plasma membrane (129) but not in liposomes, thus avoiding loss of DiO signal from liposomes (21).

Liposomes with similar composition have been used not only to characterize the phagocytic properties of microglial cells but also to assess their responsiveness to stimuli. In particular, Hashioka et al. reported that pretreatment of microglia with PS/PC (phosphatidylcholine) liposomes considerably inhibited the TNF-α, NO, and radical O_2_- production induced by Aβ/IFN-γ, suggesting that PS and PC-containing liposomes -after being phagocytosed by microglia- inhibit Aβ and interferon-γ-induced microglial activation (130). Of note, phagocytosis of PS-containing liposome has been shown to induce the secretion of anti-inflammatory mediators including prostaglandin E(2) PGE(2) (131, 132).

Thanks to their high versatility, the use of liposomes can be a very useful tool to assess microglial phagocytic functions.

### Apoptotic Neurons

Apoptotic membranes are another commonly used substrate in microglial phagocytic assays. Like in the case of beads, this type of assay can be used to investigate whether and at which extent the phagocytic machinery of microglia is functional even when synapse elimination is not properly working.

Apoptotic cells exhibit specific find-me and eat-me signals that are rapidly recognized and engulfed by phagocytes, which may or may not overlap with signals exposed at synaptic sites. Notably, phosphatidylserine externalization has been established as one of the first detectable events to occur in cells undergoing apoptosis. Clearance of apoptotic cells by phagocytes actively suppresses the initiation of inflammatory and immune responses and it is therefore fundamental for brain homeostasis (133). Moreover, apoptosis-like phenomena including caspase activation has been found locally in synapses in a process designated as “synaptic apoptosis” (134, 135).

Several protocols have been developed to study apoptotic membranes phagocytosis by microglia, describing the cellular types employed, the specific stimuli used to induce cell apoptosis and analyzing the receptors and molecular mechanisms involved. In the work by Nolte's group, cerebellar granule neurons were treated with 100 μ*M S*-nitrosocysteine to induce apoptosis, event that was confirmed by nuclear condensation and PS exposure. Primary microglial cells then were added to neurons 2 h after apoptosis induction and co-cultured for 6 h. Cultures were stained with propidium iodide (PI) (to detect apoptotic/necrotic neurons) and lectin to visualize microglia and analyzed by fluorescence microscopy (136). Zhao et al. cultured rat cortical neurons and used irradiation to induce neuronal apoptosis. Again, after propidium iodide (PI) staining, dead neurons (DNs) were exposed to microglia cultures. By fluorescence microscopy, they counted the number of DNs engulfed by each microglia, and calculated the phagocytic index, that consists in the average number of phagocytosed dead neurons (PI-DNs) within each microglia and gives a quantification of microglia phagocytic efficacy (137, 138).

Using adult microglia cultures, Butovsky et al., measured the amount of ultraviolet-irradiated (UV-irr) dead neurons engulfed by adult microglia isolated from spinal cord and cultured *in vitro*. Through IF staining they were able to quantify fluorescent dead neurons engulfed by iba1 positive cells (78). Apoptotic cellular debris can be also detected by using AnnexinV (ANXV), an innate molecule that binds with high affinity to PS-bearing membranes. As shown in a recent work performed in Drosophila, apoptotic membranes were labeled with an ANXV-conjugated fluorophore and apoptosis was induced using 10 mM cycloheximide (139).

From the point of view of the receptors or mechanisms involved in the phagocytosis of apoptotic membranes, Takahashi et al., analyzed phagocytosis of apoptotic neurons by microglia after TREM2 knockdown or overexpression. In their experimental setting, neurons were labeled by a red fluorescent membrane dye and pretreated with okaidic acid to induce apoptosis. After incubating apoptotic neurons with microglia for 1 or 24 h, phagocytosis of apoptotic membrane fragments was detected by fluorescence microscopy and flow cytometry (20, 140, 141)). Beccari et al., provided an exhaustive protocol (142) in which the authors describe a series of parameters to directly quantify in more accurate and complete way than conventionally used indirect methods, microglial apoptotic membrane phagocytosis *in vivo* and *in vitro*. In a recent work (143), the authors applied a xenogenic *in vitro* model of apoptotic cells phagocytosis to study the mechanism by which microglial phagocytosis regulates neurogenesis. Phagocytosis experiments were performed in DMEM 10% FBS to ensure the presence of complement molecules, which are related to microglial phagocytosis *in vivo* (143). Primary microglia cells were fed for different time points with SH-SY5Y cells, a human neuroblastoma cell line derived from the bone marrow, previously labeled with the membrane marker CM-DiI and treated with staurosporine to induce apoptosis. Only floating dead-cells fraction was collected from the supernatant and added to primary microglia cultures in a proportion of 1:1. Apoptotic cells were visualized and quantified by trypan blue in a Neubauer chamber. By confocal analysis, the percentage of microglia containing CM-DiI and/or DAPI inclusions along a time course was identified as actively engulfed.

### Synaptosomes

The process of synapse engulfment by microglia can be more specifically investigated using synaptosomes (SYNs), biochemically isolated structures consisting of pinched-off nerve terminals and juxtaposed postsynaptic densities. Since they maintain the molecular and biochemical features of a functioning synapse (144, 145), SYNs have been widely used by the neuroscientific community to study the synaptic structure and the functional properties of neurotransmitter release (146). In the recent years, the use of SYNs has been extended to simulate the interactions between synapses and microglia/astrocytes and test the phagocytic capacity of glial cells. To this aim, they may be used indifferently either freshly prepared or maintained as frozen (103).

To visualize their engulfment by microglia or astrocytes, SYNs can be stained with dyes sensitive to acidic pH (102–104, 147). These dyes (one of the most widely employed is pHrodo) show little or no fluorescent signal at neutral pH, while they fluoresce brightly when in acidic environments, thus allowing SYN visualization only when engulfed by acidic phagosomes. Sellgren et al. analyzed the engulfment of patient-derived SYNs by iMGLs to investigate the features of synaptic pruning in schizophrenia (SZ) patients. Phagocytosis was analyzed by both real-time imaging upon SYN labeling with a pH sensitive fluorescent dye (pHrodo) and confocal microscopy, combining the staining for pH sensitive cyanine dye and the post-synaptic marker PSD-95. Through this assay, the authors showed an increased phagocytic capacity of iMGL cells from SZ patients compared to healthy controls (103, 104).

Using a similar approach, Keaney et al. showed that the blockade of Bruton's Tyrosine Kinase (BTK), a protein involved in different processes such as B cell receptor signaling, pro-inflammatory cytokine release and phagocytosis, reduces the uptake of pHrodo-labeled SYNs by microglia (102). PHrodo labeled SYNs were also used by Madore et al., to show the relevance of poly-unsaturated omega-3 fatty acids (n-3 PUFAs) in controlling microglial phagocytosis in the developing brain. In particular, exposing microglia deriving from either n-3 deficient or n-3 sufficient mice to pHrodo labeled SYN, the authors showed that the lack of n-3 from microglia increases the phagocytic capacity of microglial cells, inducing an excessive synaptic loss (148). Recently, Evans et al., exploited pHrodo labeled SYNs in order to show that beta-adrenergic antagonists, such as metropolol, are able to significantly increase phagocytosis of primary microglia from rats, whereas beta-adrenergic agonists, such as xamoterol and isoproterenol, attenuate SYNs engulfment (149). SYNs labeled with pH sensitive dyes have been also exploited to investigate the engulfment capacity of astrocytes (147), which supported astrocyte critical role not only in trophic functions and neurotransmitters recycling, but also in synapse elimination pruning (150).

SYNs can also be stained by fluorescent dyes lacking pH sensitivity. Among these, the FM Lipophilic Styril dyes, which are able to emit fluorescence only when inserted in the outer leaflet of the plasma membrane, and are mainly used to study synaptic vesicles trafficking (103, 151). Filipello et al., exposed *Trem2* knockout (*Trem2*^−/−^) and WT primary microglia to SYNs labeled with FM1-43 dye, demonstrating a key role of TREM2 in the microglia phagocytic process. Flow cytometric analysis of CD11b^+^ microglia also positive for FM1-43 dye was successfully used to quantify SYNs engulfment (20).

Finally, SYNs can be isolated from mice expressing fluorescent markers in neuronal cells. As an example, in a recent work, researchers used SYNs derived from the brain of mice expressing red fluorescent protein (RFP)-to investigate whether microglia autophagy might be involved in synaptic pruning and be responsible for an impaired behavior (152). Using this approach, the authors showed that primary microglia lacking Atg7, which is vital for autophagy, displayed impaired degradation of (RFP)-expressing SYNs.

### Pitfalls and Hints

The exploitation of assays employing LTBs, liposomes, apoptotic membranes or synaptosomes are relatively user-friendly, yet, some specific problems can be encountered using some of these substrates. Technical details are reported in the papers specifically quoted in the chapter above. Regarding the analysis of FLB phagocytosis by microglia, it is important to precisely set both the amount of FLB used and the incubation time. Moreover, even though FLB are effectively employed without any modification (20, 21, 100, 114, 115), they cannot be considered specific phagocytic targets unless properly opsonized. Opsonization can be carried out using FBS or BSA (101, 116). However, since the amount of complement proteins like C1q and C3 in FBS and BSA is not specified, it is not possible to infer the contribution of serum to beads phagocytosis (24, 103).

Although liposomes represent a useful tool for studying targeted phagocytosis (124), an intrinsic pitfall of their preparation is the variability in their size, that is a striking characteristic to consider in the context of phagocytosis. Given UVs dimensions also influence their stability, small UVs are a good choice (124, 153). However, SUVs are not bigger than 100 nM and this could enhance the non-specific phagocytic process called macropinocytosis, or fluid phase uptake (154), which is actin-dependent and, in macrophages, it is activated by the CSF-1 (155). A basal amount of engulfed material could be thus explained by the occurrence of this process.

A more specific substrate used to analyze microglia phagocytosis is represented by synaptosomes. However, it was shown that synaptosomes expose phosphatidylserine (PS) and show caspase activation rapidly after preparation, causing alterations in assessing the phagocytic process (156).

## Experimental Settings for Investigating Synapse Elimination by Microglia

This section describes the main technical approaches which allow to directly assess the microglia ability to eliminate synaptic contacts. The chapter analyzes both *in vitro* and *in vivo* experimental settings (see Tables 1, 2).

**Table 2:** *In vivo* and *ex vivo* engulfment assays.

**Models**	**Brain areas**	**Engulfed substrates**	**Techniques adopted**	**References**
*Cx3xr1-/-* mouse	Hippocampus	SNAP25+, PSD95+ synaptic materials	Confocal microscopy; immune-gold electron microscopy	(18)
*Itgam-/-*; *C3-/-* mouse	Visual System	RGC inputs	Confocal microscopy dimensional (3D) surface volume rendering	(15)
Trem2-/- mouse	Hippocampus	PSD95+ synaptic materials	Fluorescent microscopy	(20)
Zebrafish	Spinal cord	Apoptotic neuron	Fluorescent microscopy, 3D rendering	(157)
*Mertk-/-* Mouse	Cortex	Apoptotic neuron	Time-lapse two-photon imaging	(158)
Adult microglia *ex vivo*	Cortex	Alexa-488 labeled apoptotic (dNs) or live neurons	Flow cytometry	(70)
		Amyloid beta (through the fluorescent marker Methoxy-XO4)		(159)
		Synaptic markers VGLUT1 and synaptophysin		(160)

*The table reports some of the experimental approaches used to study microglia engulfment in vivo and ex vivo in different animal models*.

### Synapse Elimination by Microglia *in vitro*

In order to directly analyze the process of synapse elimination, multicellular culture models provide several advantages (93, 161). The generation of microglial-neuron co-cultures offers flexibility in experimental design and, when exploited in concomitance with the use of cell types deriving from genetically modified mice, allows to address a variety of mechanistic questions (162–165). Furthermore, since neurons are grown separately from microglia prior the co-culture, the two cell types can be subjected to specific treatments thus allowing to test drug effects in a selected cell populations before co-culturing. In general, *in vitro* experimental systems are not able to mimic developmental stages as it occurs *in vivo*. Yet they are useful tools and, in the last years, these experimental settings have been exploited to investigate the process of synapse elimination and its molecular underpinning.

In a recent study, Lui et al., (166) focused on Progranulin (PGRN), the product of the *Grn* gene, implicated in the regulation of phagocytosis and release of pro-inflammatory cytokines from microglia and macrophages (167, 168). The authors designed a co-culture system in which wild type cortical neurons were plated at low density to allow uniform synapse development for 14 days *in vitro* (DIV14). Concurrently, microglia isolated from *Grn*^+^/^+^ or *Grn*^−^/^−^ neonatal brains were added to cortical neurons at a 1:3 microglia/neuron ratio and co-cultured for 72 h. Using a modified Sholl analysis to measure the density of synapses in the vicinity of microglia cell bodies and Imaris software to perform 3D reconstructions of confocal images, the authors quantified the amount of synaptic material within microglial phagolysosomes and demonstrated a significant increase in synaptic pruning when neurons were co-cultured with microglia isolated from mice genetically lacking *Grn*. A similar approach was used by Filipello et al., to study the role of microglial TREM2 in synapse elimination. By co-culturing microglia with hippocampal neurons at a microglia to neuron ratio of 1.5:1 for 24 h, and through the analysis of miniature excitatory post-synaptic currents (mEPSC) and dendritic spines density, the authors demonstrated that microglia are able to reduce the density of excitatory synaptic contacts *in vitro* and that microglial TREM2 is required for this process to occur (20). To better visualize neurons and spines, WT neurons were GFP-transfected at DIV 11–12 before adding microglia to the co-culture. The use of transwell inserts between the two cell types allowed to discriminate the effects of microglia that require the direct contacts with neurons.

An additional study where microglia and neurons derived from mutant or knock out mice were combined in mixed culture to investigate synapse elimination, focused on the role of PTEN, a well-recognized syndromic risk allele for autism spectrum disorder (169). Using co-cultures of primary neurons and microglia from *Pten*^*WT*/*WT*^, *Pten*^*WT*/*m*3*m*4^, or *Pten*^*m*3*m*4/*m*3*m*4^ mice in different combinations, followed by co-localization of pre- and post-synaptic markers, the authors demonstrated that *Pten*^*m*3*m*4/*m*3*m*4^ mutation results in increased microglia-dependent synaptic pruning *in vitro*. Interestingly, the largest decrease in synaptic contact density was observed when *Pten*^*m*3*m*4/*m*3*m*4^ neurons were cultured with *Pten*^*m*3*m*4/*m*3*m*4^ microglia indicating an additive effect when the mutation occurs in both cell types. A relevant technological addition of this study is the setting of a protocol which allows co-culturing microglia and neurons for a week in a microglia/neuron ratio 1:1 (i.e., a longer time compared to the generally used general protocols).

The co-culture setting allows to test pharmacological or experimental treatments which reduce microglial phagocytic ability. Inhibition of synapse phagocytosis *in vitro* was recently demonstrated upon the exposure of hippocampal neurons to ANXV, an innate molecule that binds phosphatidylserine- bearing membranes with high affinity, 15 min before co-culturing them with microglia. ANXV, by cloaking externalized PS, prevents its recognition by microglial TREM2 and prevents synapse elimination, as demonstrated by the lack of dendritic spine density and mEPSC frequency reduction. A similar approach was taken in another recent work, where microglial cells were exposed to different treatments before being added to neuronal cultures (100). Specifically, Allendorf et al., demonstrated that LPS, fibrillar Aβ, phorbol 12-myristate 13-acetate (PMA) or rTAU protein induced removal of sialic acid residues in microglial cells. This resulted in an enhanced microglia ability to phagocytose neuronal components. Of note neuronal phagocytosis was inhibited by a blocking antibody against CD11b/CR3 (100).

Besides co-cultures of murine microglia and neurons, recent studies took advantage of the use of human cells. In a very interesting paper, Sellgren et al. developed and validated a high-throughput method for modeling synaptic pruning *in vitro*, using cells derived from SZ patients or healthy subjects (103). Specifically, the authors employed iPSC-derived-microglia like cells and iPSC-derived neurons, the latter generated from an inducible neurogenin 2 (NGN2) expressing stable NPC lines. After 21 days of neural differentiation, mature iMGLs derived from monocytes were added to neurons for 48 h. iMGLs, maintained under serum-free *in vitro* conditions, were found to engulf synapses from iPSC-derived neural cultures, as assessed by live imaging of iPSC-derived neurons stained for Alexa Fluor 488-phalloidin and by measuring PSD-95 engulfment. Using this asset, the authors demonstrated a significantly higher, complement-dependent, uptake of synaptic structures when cells from SZ patients were employed (103).

### Synapse Elimination by Microglia *in vivo*

Since Ito et al. in 1998 isolated and identified a novel gene “the *iba1* gene” specifically expressed in microglia, traditionally, Iba-1 antibodies have been used to label/stain microglia using immunohistochemistry (170). Confocal laser scanning microscopy is frequently used to image fluorescently labeled microglia in tissue sections (fixed), retinal whole mounts (fixed or fresh) and organotypic brain slices (fresh) to investigate microglial density, morphology, distribution, and dynamic interactions with different cell types (171, 172).

In the last decades, thanks to the advancement of high resolution live microscopy techniques, Iba1-positive microglia have been characterized as highly motile cells, extending and retracting their processes as they survey the microenvironment in the healthy brain (173). Both pre-synaptic boutons and postsynaptic spines have been shown to be contacted by microglial processes (6, 174). In the visual cortex, the microglia-synapse contacts were examined in closer resolution using 3D reconstruction serial electron microscopy (6). This study revealed that, in addition to pre- and postsynaptic specializations, microglial processes also contacted peri-synaptic astrocytes and the synaptic cleft.

Subsequently, the close microglia-synapse contacts appeared to result in the shaping, or re-wiring, of neuronal circuits by phagocytosis of synaptic materials. The phagocytic properties of microglia have been extensively analyzed through different microscopy-based approaches: confocal imaging, electron microscopy, two-photon microscopy and lightsheet microscopy (15, 173, 175–177). These techniques allow to visualize and quantify, in a very reliable manner, the material engulfed by microglia in the brain, generating a clear picture of the phagocytic process in specific time windows. 3D reconstruction of the phagocyte and its intracellular structures (e.g., phagolysosomes and other intracellular organelles) by softwares like Imaris, ilastik [(178); 1.3.2] and CellProfiler [(179); v3.0] has been successfully used to generate very detailed images of phagocytic microglia and to quantify the material internalized.

Two milestones articles first demonstrated, by electron microscopy and super-resolution confocal microscopy, the presence of pre- and post-synaptic structures inside microglial phagolysosomes in different brain regions (mouse visual system and hippocampus) during critical periods of synaptic refinement. In particular, in 2011, Paolicelli et al. spotted synaptic material inside microglia, providing the demonstration that these cells play an active role in pruning synapses. Specific presynaptic (SNAP25) and postsynaptic (PSD95) proteins were identified inside microglial processes following synaptic contacts, by confocal or immune-gold electron microscopy, respectively (18). Furthermore, disrupting the fractalkine (Cx3cl1/Cx3cr1)-mediated communication between microglia and neurons in an otherwise healthy mouse, resulted in brain circuits persisting as immature into adulthood (18, 180, 181). In 2012, the Stevens' lab at Boston Children's Hospital, found that, in the newborn mouse visual system, microglia can engulf synapses in the lateral geniculate nucleus (LGN) through a process mediated by both complement and neuronal activity. Using Cholera Toxin B Subunit (CTB) injections in *Cx3cr1* ^gfp/+^ mice, in which microglia express GFP, the authors elegantly showed for the first time, by 3D reconstructions that microglia contain engulfed RGC inputs. By either silencing or promoting neuronal activity in one eye using TTX or forskolin, respectively, they further showed that microglia selectively prune the weaker inputs. Notably, by examining microglial engulfment in C3 mutants and C3-receptor mutants, Schafer et al. showed that this process critically relies on the complement cascade. Of note, impaired microglial engulfment in both these mutants correlated with long-lasting defects in the segregation of ipsi- and contralateral RGC inputs in the dLGN, with an increase in synaptic densities (15). To confirm that inputs are in fact phagocytosed by microglia, Schafer et al. introduced a staining of *in situ* microglia for the phagolysosomal marker CD68, performing the subsequent colocalization with synaptic materials. Only the synaptic material internalized in CD68-positive phagolysosomal structures was considered for the analysis. A few years later, the same group published a detailed methodology for imaging and quantitatively measuring engulfment using confocal microscopy combined with 3D surface volume rendering, a method which is widely used by the scientific community (182).

Still today these two papers represent the landmark for researchers interested in studying microglia-mediated synapse elimination *in vivo*. Indeed, most if not all the subsequent studies heavily relied on the methods introduced by these pioneering works. Filipello et al. (20), used the same protocol of engulfment analysis and quantification proposed by Schafer et al. to describe the role of TREM2 in regulating synapse phagocytosis during hippocampal development. The same approaches were used to demonstrate the role of CD47, a transmembrane immunoglobulin superfamily protein that directly inhibits phagocytosis by binding to its receptor, SIRPα, thus behaving as a “don't eat me” signal during postnatal development (22). With the aim to detect the phagocytosis of a different substrate, a similar approach was also taken by Cignarella et al. who analyzed myelin engulfment and degradation by microglia in the cuprizone model of brain demyelination. By confocal analysis and subsequent 3D reconstruction, the authors showed that a TREM2 agonistic antibody enhanced myelin uptake and degradation, resulting in accelerated myelin debris removal by microglia. Again, 3D reconstruction by the Imaris software of CD68 structures inside Iba1-positive microglia containing dMBP-positive myelin debris, was used as a consolidated method of analysis (183).

Using time-lapse imaging, Weinhard et al., recently reported that, rather than removing the whole synaptic structure, microglia prune presynaptic structures through a selective partial phagocytic process termed trogocytosis, or “nibbling.” The authors studied microglia “nibbling” on presynaptic structures of neurons in organotypic tissue culture, an *ex vivo* model that preserves tissue architecture important for microglia physiology and offers the advantages of a tissue-relevant context effective in studying the synaptic elimination processes. Subsequent analysis of fixed hippocampal tissue from postnatal day 15 (P15) mice using quantitative confocal microscopy as well as correlative light and electron microscopy, revealed that microglia only capture small (<1 um) presynaptic components though a process which involves the “sinking” of presynaptic structures into the microglial cytoplasm prior to membrane closure. Conversely, pseudopodia, a hallmark of phagocytosis, were not observed (17). Further lines of investigation are expected to provide additional insights into the precise mechanisms by which microglia remove and digest synaptic contacts.

### Facs-Based Microglia Phagocyitc Assays *ex vivo*

The analyses described in the previous paragraphs rely on the use of *in vitro* microglia, prepared as described in Microglia Cell Lines, Primary Newborn Microglia, and Human Microglia-Like Cells sections. However, similar assays can also be performed taking advantage of microglia freshly isolated from the adult or juvenile brain and analyzed right away (see Primary Adult Microglia chapter). The latter setting maintains closer features to those of the same cells when present in brain environment, despite of the isolation process and the consequent manipulation. In 2007, Biber's group showed the possibility to isolate microglia from specific brain regions (optic nerve, striatum, hippocampus, cerebellum, spinal cord, cortex) and to quantify the amount of fluorescent microspheres phagocytosis by confocal microscopy (62).

In the recent years, the use of flow cytometry has implemented microscopy techniques thus becoming a very useful approach to dissect the phagocytic properties of microglia not only in *in vitro* assays but also using freshly isolated microglia *ex vivo*. This strategy was successfully used by Krasemann et al., who identified a role for apolipoprotein E (APOE) in regulating a subset of microglia, exhibiting a common neurodegenerative- associated phenotype (MGnD). To determine the mechanisms through which MGnD were induced during neurodegeneration, they injected Alexa-488 labeled apoptotic (dead, dNs) or live neurons (Ns) into the cortex and hippocampus of naïve mice. In parallel, they also injected fluorescent E. coli or zymosan as a control. By gating the CD11b^+^ CD45^low^ population they were able to distinguish the phagocytic cells that internalized 488-labeled apoptotic neurons (CD11b^+^ CD45^low^ dNs-Alexa 488^+^) vs. non-phagocytic microglia (CD11b^+^ CD45^low^ dNs-Alexa 488^−^) (70).

A similar approach was used by Tejera and Heneka who showed in detail how to analyze Aβ phagocytosis by flow cytometry using microglia freshly isolated from adult mice. Mice were intraperitoneally injected with the Aβ fluorescent marker Methoxy-XO4, and microglia were isolated through a Percoll gradient and directly analyzed by FACS. The CD11b^+^CD45^low^ population, also positive for Methoxy-XO4, represented microglia phagocytosing Aβ (159). Using a different strategy, Levey's group validated a rapid flow cytometric assays to test phagocytic capacity of acutely isolated CNS mononuclear phagocytes (MPs). MPs were isolated through a Percoll gradient and subsequently incubated with macroparticle and fibrillar Aβ42 (fAβ42). Flow cytometric analysis revealed distinct phagocytic capacities of CD11b^+^CD45^low^ and CD11b^+^CD45^high^ cells both in physiological condition and in disease models (184).

The use of mass cytometry (CyTOF), a technique that combines flow cytometry with mass spectrometry, has enabled a high-dimensional analysis of cell surface markers, signaling molecules and cytokines in brain myeloid cells at the single-cell level (185–187). Because the method is largely unhampered by interference from spectral overlap, it allows for the detection of considerably more simultaneous parameters than does traditional flow cytometry. This has facilitated the understanding of phenotypic diversity of mouse and human macrophages *in vitro* and *in vivo* (188, 189). Interestingly, different macrophage phenotypes were found to have different phagocytic activities. In 2019, Schulz et al., created a functional assay to assess phagocytic activity of macrophages by mass cytometry. This method combines an in-depth phenotypic characterization of macrophages based on the expression of 36 protein markers with an analysis of biological function. The authors assessed the abilities of macrophages activated *in vitro* under different conditions to phagocytose bacteria and cancer cells. By correlating the phagocytic activity with markers expression of single cells, they defined characteristic signatures preferentially associated with phagocytosis of specific targets. This strategy can be also applied to better understand and link cell phenotype to phagocytic function in microglia in health and disease (106).

### Pitfalls and Hints

The study of synapse elimination using co-cultures of neurons and microglia requires specific attention especially in relation to the establishment of the adequate co-culture conditions. In particular, defining the optimal density of microglial cells and the neuron/microglia ratio represents the most critical issue. The optimal ratio may vary depending on the experimental design and should be established accordingly. Another limitation to be considered is the limited time window (24–72 h) during which the microglia-neuron model can be maintained in co-culture. This limitation, which results from the fact that the two different cell types prefer different culture conditions (56), discourage the setting of experiments addressing processes which develop in the long term. The limited time-scale of this model is due to the negative effect of the continuous presence of microglia on the overall health of the neurons and to the fact that the culture media contains a high concentration of serum used to support the microglia, likely causing the microglia to be in an already activated state.

To overcome this issue, recently, it has been developed a tri-culture system consisting of neurons, astrocytes, and microglia. Primary rat cortical cells were maintained in a serum-free culture media developed to support all three cell types. It has been demonstrated that adding astrocytes in the culture system ameliorates neurons conditions. This “tri-culture” system can be maintained for at least 14 days *in vitro* (DIV), without any negative effect of the continuous presence of microglia on the overall health of the neurons (93).

Regarding the *in vivo* studies, one of the major risks associated with the study of microglia *in vivo*, is that manipulation of the CNS tissues (as an example, during brain slices preparation) can lead to tissue injury and subsequent microglia activation. To solve this issue, tissue clearing techniques coupled with light sheet microscopy can be used to visualize microglia within intact transparent CNS tissues. Although, so far, this technique has not been used to study synaptic pruning, it could be relevant in the future. Indeed, besides allowing an unbiased global investigation, the method will eliminate the need to perform histological sectioning [methods and applications reviewed in (190)].

Moreover, it needs to be considered that iba1 antibodies which have been traditionally used to label microglia *in situ*, also recognize border-associated macrophages (BAMs) as well as subsets of peripheral myeloid cells. The possibility to differentiate microglia from BAMs, which reside within the meninges, choroid plexus and brain perivascular spaces as well as from circulating myeloid cells that infiltrate the CNS during neuroinflammation, is therefore mandatory. More recent studies have focused on identifying microglia-specific markers that can reliably distinguish microglia from other leukocytes, both in healthy conditions and disease. Given the range of markers and antibodies that can be used to identify microglia, the choice of targets needs to be carefully considered for each scientific question. Under this respect, recently described reporter mice have taken advantage of microglia-specific signature genes, including Tmem119eGFP (140), Tmem119TdTomato (141), Sall1GFP (142), and HexbTdTomato (159) mice are knock-in strains in which the expression of fluorescent reporter proteins is largely restricted to microglia. Another critical point when studying microglia *in vivo* is most of the confocals microscopes have limited imaging depth and require therefore the specimens to be sectioned (brain) or microdissected (retina). Also, image acquisition can be slow and Photobleaching of tissue can occur, while fixation may affect MG morphology. Finally, although modern microscopy provides a qualitative appraisal of synaptic proteins inside microglia, yet they have some drawbacks for a fast and unbiased quantification. In particular, the spatial resolution of confocal microscopy may be insufficient to resolve microglial and synaptic structures when they are less than few hundreds of nanometers apart from each other (17) [see (184) for an exhaustive review of several confocal, multiscale imaging methods for brain research]. Further, although limitations due to the fact that penetration of infrared light is limited to 1,000 μm in depth from the surface (191), *in vivo* two-photon excitation microscopy enabled direct measurement of synapse turnover in mice at postnatal 2 and 3 weeks and obtained the reliable data of spine turnover in neocortical areas (192). Synapse turnover in the hippocampus and other subcortical areas can be measured by endoscope technology, although this technique is less reliable than the two-photon imaging, mainly due to the lower resolution (192).

Given the technical limitations, the development of alternative approaches is currently in high demand (133). New technologies that could provide important advantages are holographic microscopy which brings the resolution of electron microscopy to the order of the Armstrong, and multi-isotope imaging mass spectrometry. The latter technique also allows to image and quantify molecules and presents a great potential for identifying new molecular targets in neuroimmunological field (145).

The analysis of freshly isolated microglia by FACS-based phagocytic assays *ex vivo* may pose a few specific problems. As already mentioned, it is critical to choose protocols generating freshly isolated microglia from the adult brain that avoid hyper-activation and stress of this type of cells. Again, flow cytometers need to be up to date, and FACS lasers should be often calibrated and constantly maintained by specialists at the FACS facilities. The combination of antibodies used to stain microglia and to detect the phagocytic material need to be chosen taking into account fluorophore emission/excitation spectra overlapping and the subsequent compensation. It is always necessary to add the proper isotype controls to the staining panel to discriminate unspecific signal. Negative and positive controls and cells deriving from mice knockout for the specific gene of interest should be run in parallel when analyzing signal/proteins that have not been described before. Importantly, the gating strategy used to select CD11b^+^ CD45^low^ microglia should be carefully chosen taking into account that other immune cells also positive for those markers are present in the brain parenchima and meninges. Finally, when choosing antibodies to specifically target microglia (i.e., Tmem119, P2ry12) it is important to consider that during pathological /inflammatory conditions these molecules can change being down or upregulated, therefore making it necessary to revise the gating strategy and the antibodies panel.

## Conclusions

In the last years, several novel techniques and approaches have been introduced which have significantly advanced the study of how microglia cells, at specific stages of brain development, perform engulfment and elimination of neuronal and synaptic components. Phagocytic assays employing liposomes, synaptosomes or apoptotic membranes allow the dissection of the molecular and lipidic components that direct the engulfment process. Co-cultures of neurons and microglia derived from WT or genetically modified mouse models provide the possibility to successfully assess, by different methods, the molecular requirements and the functional consequences of the synapse elimination process. While these methods provide settings suited to easily investigate the mechanistic aspects of the process of microglia-mediated phagocytosis, they suffer from the major problem that isolated microglia do not maintain the phenotypic and functional features they have in the brain. The *in vitro* assays need therefore to be combined with analysis in brain sections or using microglia freshly isolated from the adult or juvenile brain and immediately analyzed, which allows to maintain closer features to those of the same cells present in brain environment. The use of flow cytometry has implemented confocal and electron microscopy techniques, revealing as a very useful approach.

Finally, the possibility to generate microglia-like cells from human embryonic stem cells or by the reprogramming of adult cells into induced pluripotent stem cells is providing new, important possibilities to investigate the process of neuronal and synaptic phagocytosis employing material derived from human patients. It is expected that these methods will be soon implemented by the possibility of incorporating the appropriate number of microglia-like cells derived from human embryonic stem cells into brain organoids, in order to obtain a cell type ratio comparable to that of the human brain and allowing at the same time the microglia differentiation in a 3-dimensional structure. Together with the combined use of high resolution microscopy, FACS and mass cytometry analysis, we can expect that these approaches will represent a further step toward a deeper comprehension of the process of synapse elimination in healthy or diseased contexts.

## Author Contributions

RM, FF, and MM designed the review outline. All the authors contributed to writing and designing the scheme.

## Conflict of Interest

The authors declare that the research was conducted in the absence of any commercial or financial relationships that could be construed as a potential conflict of interest.

## Abbreviations

Atg7: Autophagy Related 7
C1q: Complement Component 1q
C3: Complement Component 3
CD11b: Cluster of Differentiation 11b
CD200: Cluster of Differentiation 200
CD206: Cluster of Differentiation 206
CD45: Cluster of Differentiation 45
CD47: Cluster of Differentiation 47
CD68: Cluster of Differentiation 68
CD86: Cluster of Differentiation 86
CSF-1: macrophage colony-stimulating factor 1
CX3CL1: CX3C- chemokine ligand 1
Cx3CR1: C-X3-C Motif Chemokine Receptor 1
EZH2: Enhancer of Zeste Homolog 2
FBS: Fetal Bovine Serum
GM-CSF: macrophage colony-stimulating factor 1
GRN: Granulin
IBA1: Ionized Calcium-Binding Adapter 1
IFNγ: Interferon γ
IGF-1: Insulin Like Growth Factor 1
IL- 10: Interleukin 10
IL-1β: Interleukin 1 β
IL-34: Interleukin-34
IL-4: Interleukin 4
iNOS: Cytokine-inducible Nitric Oxidase Synthase
LPS: Lipopolysaccharide
PPARγ: Peroxisome Proliferator-activated receptor γ
PRC2: Polycomb Repressive Complex 2
PSD-95: Post synaptic Density Protein 95
PTEN: Phosphatase and Tensin Homolog
SIRPα: Signal Regulatory Protein α
SNAP25: Synaptosomal Associated Protein
SZ: Schizophrenia
TDP-43: TAR DNA-binding Protein
Tgfbr1: Transforming Growth Factor β receptor 1
Tgfbr2: Transforming Growth Factor β receptor 2
TNFα: Tumor Necrosis Factor α
TREM2: Triggering Receptor Expressed on Myeloid Cells 2
TTX: Tetrodotoxin
vGLUT1: Vescicular Glutamate Transporter 1
SR-A: Scavenger Receptor A.

## References

[1] Allen NJ Lyons DA. Glia as architects of central nervous system formation and function. Science. (2018) 362:181–5. 10.1126/science.aat0473 30309945 PMC6292669

[2] Reemst K Noctor SC Lucassen PJ Hol EM. The indispensable roles of microglia and astrocytes during brain development. Front Hum Neurosci. (2016) 10:1–28. 10.3389/fnhum.2016.00566 27877121 PMC5099170

[3] Ginhoux F Greter M Leboeuf M Nandi S See P Gokhan S. Fate mapping analysis reveals that adult microglia derive from primitive macrophages. Science. (2010) 330:841–5. 10.1126/science.1194637 20966214 PMC3719181

[4] Schafer DP Stevens B. Microglia function in central nervous system development and plasticity. Cold Spring Harb Perspect Biol. (2015) 7:1–8. 10.1101/cshperspect.a020545 26187728 PMC4588063

[5] Wake H Moorhouse AJ Jinno S Kohsaka S Nabekura J. Resting microglia directly monitor the functional state of synapses *in vivo* and determine the fate of ischemic terminals. J Neurosci. (2009) 29:3974–80. 10.1523/JNEUROSCI.4363-08.2009 19339593 PMC6665392

[6] Tremblay ME Lowery RL Majewska AK. Microglial interactions with synapses are modulated by visual experience. PLoS Biol. (2010) 8:e1000527. 10.1371/journal.pbio.1000527 21072242 PMC2970556

[7] Miyamoto A Wake H Ishikawa AW Eto K Shibata K Murakoshi H. Microglia contact induces synapse formation in developing somatosensory cortex. Nat Commun. (2016) 7:12540. 10.1038/ncomms12540 27558646 PMC5007295

[8] Parkhurst CN Yang G Ninan I Savas JN Yates JR III Lafaille JJ. Microglia promote learning-dependent synapse formation through brain-derived neurotrophic factor. Cell. (2013) 155:1596–609. 10.1016/j.cell.2013.11.030 24360280 PMC4033691

[9] Hammond TR Dufort C Dissing-Olesen L Giera S Young A Wysoker A. Single-Cell RNA sequencing of microglia throughout the mouse lifespan and in the injured brain reveals complex cell-state changes. Immunity. (2019) 50:253–71.e6. 10.1016/j.immuni.2018.11.004 30471926 PMC6655561

[10] Feinberg I. Schizophrenia: caused by a fault in programmed synaptic elimination during adolescence? J Psychiatr Res. (1982) 17:319–34. 10.1016/0022-3956(82)90038-3 7187776

[11] Huttenlocher PR. Synaptic density in human frontal cortex - developmental changes and effects of aging. Brain Res. (1979) 163:195–205. 10.1016/0006-8993(79)90349-4 427544

[12] Shatz CJ. The prenatal development of the cat's retinogeniculate pathway. J Neurosci. (1983) 3:482–99. 10.1523/JNEUROSCI.03-03-00482.1983 6402566 PMC6564557

[13] Sretavan D Shatz CJ. Prenatal development of individual retinogeniculate axons during the period of segregation. Nature. (1984) 308:845–8. 10.1038/308845a0 6201743

[14] Li Y Du X-F Liu C-S Wen Z-L Du J-L. Reciprocal regulation between resting microglial dynamics and neuronal activity *in vivo*. Dev Cell. (2012) 23:1189–202. 10.1016/j.devcel.2012.10.027 23201120

[15] Schafer DP Lehrman EK Kautzman AG Koyama R Mardinly AR Yamasaki R. Microglia sculpt postnatal neural circuits in an activity and complement-dependent manner. Neuron. (2012) 74:691–705. 10.1016/j.neuron.2012.03.026 22632727 PMC3528177

[16] Stevens B Allen NJ Vazquez LE Howell GR Christopherson KS Nouri N. The classical complement cascade mediates CNS synapse elimination. Cell. (2007) 131:1164–78. 10.1016/j.cell.2007.10.036 18083105

[17] Weinhard L Di Bartolomei G Bolasco G Machado P Schieber NL Neniskyte U. Microglia remodel synapses by presynaptic trogocytosis and spine head filopodia induction. Nat Commun. (2018) 9:1228. 10.1038/s41467-018-03566-5 29581545 PMC5964317

[18] Paolicelli RC Bolasco G Pagani F Maggi L Scianni M Panzanelli P. Synaptic pruning by microglia is necessary for normal brain development. Science. (2011) 333:1456–8. 10.1126/science.1202529 21778362

[19] Ulland TK Colonna M. TREM2 - a key player in microglial biology and Alzheimer disease. Nat Rev Neurol. (2018) 14:667–75. 10.1038/s41582-018-0072-1 30266932

[20] Filipello F Morini R Corradini I Zerbi V Canzi A Michalski B. The microglial innate immune receptor TREM2 is required for synapse elimination and normal brain connectivity. Immunity. (2018) 48:979–91.e8. 10.1016/j.immuni.2018.04.016 29752066

[21] Scott-Hewitt N Perrucci F Morini R Erreni M Mahoney M Witkowska A. Local externalization of phosphatidylserine mediates developmental synaptic pruning by microglia. EMBO J. (2020) 39:e105380. 10.15252/embj.2020105380 32657463 PMC7429741

[22] Lehrman EK Wilton DK Litvina EY Welsh CA Chang ST Frouin A. CD47 protects synapses from excess microglia-mediated pruning during development. Neuron. (2018) 100:120–34.e6. 10.1016/j.neuron.2018.09.017 30308165 PMC6314207

[23] Salter MW Stevens B. Microglia emerge as central players in brain disease. Nat Med. (2017) 23:1018–27. 10.1038/nm.4397 28886007

[24] Hong S Beja-Glasser VF Nfonoyim BM Frouin A Li S Ramakrishnan S. Complement and microglia mediate early synapse loss in Alzheimer mouse models. Science. (2016) 352:712–6. 10.1126/science.aad8373 27033548 PMC5094372

[25] Dejanovic B Huntley MA De Mazière A Meilandt WJ Wu T Srinivasan K. Changes in the synaptic proteome in tauopathy and rescue of tau-induced synapse loss by C1q antibodies. Neuron. (2018) 100:1322–36.e7. 10.1016/j.neuron.2018.10.014 30392797

[26] Ulrich JD Ulland TK Colonna M Holtzman DM. Elucidating the role of TREM2 in Alzheimer's disease. Neuron. (2017) 94:237–48. 10.1016/j.neuron.2017.02.042 28426958

[27] Han MH Lundgren DH Jaiswa S Chao M Graham KL Garris CS. Janus-like opposing roles of CD47 in autoimmune brain inflammation in humans and mice. J Exp Med. (2012) 209:1325–34. 10.1084/jem.20101974 22734047 PMC3405500

[28] Janda E Boi L Carta AR. Microglial phagocytosis and its regulation: a therapeutic target in parkinson's disease? Front Mol Neurosci. (2018) 11:144. 10.3389/fnmol.2018.00144 29755317 PMC5934476

[29] Blasi E Barluzzi R Bocchini V Mazzolla R Bistoni F. Immortalization of murine microglial cells by a v-raf/v-myc carrying retrovirus. J Neuroimmunol. (1990) 27:229–37. 10.1016/0165-5728(90)90073-V 2110186

[30] Bignami A Eng LF Dahl D Uyeda CT. Localization of the glial fibrillary acidic protein in astrocytes by immunofluorescence. Brain Res. (1972) 43:429–35. 10.1016/0006-8993(72)90398-8 4559710

[31] Mazaheri F Snaidero N Kleinberger G Madore C Daria A Werner G. TREM2 deficiency impairs chemotaxis and microglial responses to neuronal injury. EMBO Rep. (2017) 18:1186–98. 10.15252/embr.201743922 28483841 PMC5494532

[32] Stansley B Post J Hensley K. A comparative review of cell culture systems for the study of microglial biology in Alzheimer's disease. J Neuroinflamm. (2012) 9:115. 10.1186/1742-2094-9-115 22651808 PMC3407712

[33] Boza-Serrano A Reyes JF Rey NL Leffler H Bousset L Nilsson U. The role of Galectin-3 in α-synuclein-induced microglial activation. Acta Neuropathol Commun. (2014) 2:156. 10.1186/PREACCEPT-1285543917141325 25387690 PMC4236422

[34] Kopec KK Carroll RT. Alzheimer's beta-amyloid peptide 1-42 induces a phagocytic response in murine microglia. J Neurochem. (1998) 71:2123–31. 10.1046/j.1471-4159.1998.71052123.x 9798938

[35] Righi M Mori L De Libero G Sironi M Biondi A Mantovani A. Monokine production by microglial cell clones. Eur J Immunol. (1989) 19:1443–8. 10.1002/eji.1830190815 2789141

[36] Ferrari D Villalba M Chiozzi P Falzoni S Ricciardi-Castagnoli P Di Virgilio F. Mouse microglial cells express a plasma membrane pore gated by extracellular ATP. J Immunol. (1996) 156:1531–9. 8568257

[37] Hickman SE Allison EK El Khoury J. Microglial dysfunction and defective beta-amyloid clearance pathways in aging Alzheimer's disease mice. J Neurosci. (2008) 28:8354–60. 10.1523/JNEUROSCI.0616-08.2008 18701698 PMC2597474

[38] Walker WS Gatewood J Olivas E Askew D Havenith CE. Mouse microglial cell lines differing in constitutive and interferon-gamma-inducible antigen-presenting activities for naive and memory CD4+ and CD8+ T cells. J Neuroimmunol. (1995) 63:163–74. 10.1016/0165-5728(95)00146-8 8550814

[39] Timmerman R Burm SM Bajramovic JJ. An overview of *in vitro* methods to study microglia. Front Cell Neurosci. (2018) 12:242. 10.3389/fncel.2018.00242 30127723 PMC6087748

[40] Alliot F Marty M-C Cambier D Pessac B. A spontaneously immortalized mouse microglial cell line expressing CD4. Dev Brain Res. (1996) 95:140–3. 10.1016/0165-3806(96)00101-0 8873987

[41] Takenouchi T Ogihara K Sato M Kitani H. Inhibitory effects of U73122 and U73343 on Ca2+ influx and pore formation induced by the activation of P2X7 nucleotide receptors in mouse microglial cell line. Biochim Biophys Acta. (2005) 1726:177–86. 10.1016/j.bbagen.2005.08.001 16122875

[42] Sousa C Biber K Michelucci A. Cellular and molecular characterization of microglia: a unique immune cell population. Front Immunol. (2017) 8:198. 10.3389/fimmu.2017.00198 28303137 PMC5332364

[43] Tao X Li N Liu F Hu Y Liu J Zhang Y-M. *In vitro* examination of microglia-neuron crosstalk with BV2 cells, and primary cultures of glia and hypothalamic neurons. Heliyon. (2018) 4:e00730. 10.1016/j.heliyon.2018.e00730 30148218 PMC6106694

[44] Henn A Lund S Hedtjärn M Schrattenholz A Pörzgen P Leist M. The suitability of BV2 cells as alternative model system for primary microglia cultures or for animal experiments examining brain inflammation. ALTEX. (2009) 25:83–94. 10.14573/altex.2009.2.83 19565166

[45] Lorsch JR Collins FS Lippincott-Schwartz J. Cell biology. Fixing problems with cell lines. Science. (2014) 346:1452–53. 10.1126/science.1259110 25525228 PMC5101941

[46] Das A Chai JC Kim SH Park KS Lee YS Jung KH. Dual RNA sequencing reveals the expression of unique transcriptomic signatures in lipopolysaccharide-induced BV-2 microglial cells. PLoS ONE. (2015) 10:e0121117. 10.1371/journal.pone.0121117 25811458 PMC4374676

[47] Das A Kim SH Arifuzzaman S Yoon T Chai JC Lee YS. Transcriptome sequencing reveals that LPS-triggered transcriptional responses in established microglia BV2 cell lines are poorly representative of primary microglia. J Neuroinflamm. (2016) 13:182. 10.1186/s12974-016-0644-1 27400875 PMC4940985

[48] Constantinescu CS Goodman DB Hilliard B Wysocka M Cohen JA. Murine macrophages stimulated with central and peripheral nervous system myelin or purified myelin proteins release inflammatory products. Neurosci Lett. (2000) 287:171–4. 10.1016/S0304-3940(00)01184-8 10863022

[49] Proudfoot AEI de Souza ALS Muzio V. The use of chemokine antagonists in EAE models. J Neuroimmunol. (2008) 198:27–30. 10.1016/j.jneuroim.2008.04.007 18550179

[50] Butovsky O Jedrychowski MP Moore CS Cialic R Lanser AJ Gabriely G. Identification of a unique TGF-β-dependent molecular and functional signature in microglia. Nat Neurosci. (2014) 17:131–43. 10.1038/nn.3599 24316888 PMC4066672

[51] Lee J-K Tansey MG. Microglia isolation from adult mouse brain. Methods Mol Biol. (2013) 1041:17–23. 10.1007/978-1-62703-520-0_3 23813365 PMC4145600

[52] Harms AS Tansey MG. Isolation of murine postnatal brain microglia for phenotypic characterization using magnetic cell separation technology. Methods Mol Biol. (2013) 1041:33–9. 10.1007/978-1-62703-520-0_5 23813367 PMC4141886

[53] Lian H Roy E Zheng H. Protocol for primary microglial culture preparation. Bio Protocol. (2016) 6:e1989. 10.21769/BioProtoc.1989 29104890 PMC5669279

[54] Holtman IR Skola D Glass CK. Transcriptional control of microglia phenotypes in health and disease. J Clin Invest. (2017) 127:3220–9. 10.1172/JCI90604 28758903 PMC5669536

[55] Amit I Winter DR Jung S. The role of the local environment and epigenetics in shaping macrophage identity and their effect on tissue homeostasis. Nat Immunol. (2016) 17:18–25. 10.1038/ni.3325 26681458

[56] Bohlen CJ Bennett FC Tucker AF Collins HY Mulinyawe SB Barres BA. Diverse requirements for microglial survival, specification, and function revealed by defined-medium cultures. Neuron. (2017) 94:759–73.e8. 10.1016/j.neuron.2017.04.043 28521131 PMC5523817

[57] Hayes GM Woodroofe MN Cuzner ML. Characterisation of microglia isolated from adult human and rat brain. J Neuroimmunol. (1988) 19:177–89. 10.1016/0165-5728(88)90001-X 3410964

[58] Sedgwick JD Schwender S Imrich H Dörries R Butcher GW ter Meulen V. Isolation and direct characterization of resident microglial cells from the normal and inflamed central nervous system. Proc Natl Acad Sci USA. (1991) 88:7438–42. 10.1073/pnas.88.16.7438 1651506 PMC52311

[59] Ford AL Goodsall AL Hickey WF Sedgwick JD. Normal adult ramified microglia separated from other central nervous system macrophages by flow cytometric sorting. Phenotypic differences defined and direct *ex vivo* antigen presentation to myelin basic protein-reactive CD4+ T cells compared. J Immunol. (1995) 154:4309–21. 7722289

[60] De Groot CJ Montagne L Janssen I Ravid R Van Der Valk P Veerhuis R. Isolation and characterization of adult microglial cells and oligodendrocytes derived from postmortem human brain tissue. Brain Res Brain Res Protoc. (2000) 5:85–94. 10.1016/S1385-299X(99)00059-8 10719269

[61] Cardona AE Huang D Sasse ME Ransohoff RM. Isolation of murine microglial cells for RNA analysis or flow cytometry. Nat Protoc. (2006) 1:1947–51. 10.1038/nprot.2006.327 17487181

[62] de Haas AH Boddeke HWGM Brouwer N Biber K. Optimized isolation enables *ex vivo* analysis of microglia from various central nervous system regions. Glia. (2007) 55:1374–84. 10.1002/glia.20554 17661344

[63] Nikodemova M Watters JJ. Efficient isolation of live microglia with preserved phenotypes from adult mouse brain. J Neuroinflamm. (2012) 9:147. 10.1186/1742-2094-9-147 22742584 PMC3418565

[64] Yip PK Kaan TKY Fenesan D Malcangio M. Rapid isolation and culture of primary microglia from adult mouse spinal cord. J Neurosci Methods. (2009) 183:223–37. 10.1016/j.jneumeth.2009.07.002 19596375

[65] Garcia JA Cardona SM Cardona AE. Isolation and analysis of mouse microglial cells. Curr Protoc Immunol. (2014) 104:14.35.1–15. 10.1002/0471142735.im1435s104 24510618 PMC3980480

[66] Joseph B Venero JL. A brief overview of multitalented microglia. Methods Mol Biol. (2013) 1041:3–8. 10.1007/978-1-62703-520-0_1 23813363

[67] Bennett ML Bennett FC Liddelow SA Ajami B Zamanian JL Fernhoff NB. New tools for studying microglia in the mouse and human CNS. Proc Natl Acad Sci USA. (2016) 113:E1738–46. 10.1073/pnas.1525528113 26884166 PMC4812770

[68] Olah M Raj D Brouwer N De Haas AH Eggen BJL Den Dunnen WFA. An optimized protocol for the acute isolation of human microglia from autopsy brain samples. Glia. (2012) 60:96–111. 10.1002/glia.21251 21989594

[69] Sierra A Gottfried-Blackmore AC McEwen BS Bulloch K. Microglia derived from aging mice exhibit an altered inflammatory profile. Glia. (2007) 55:412–24. 10.1002/glia.20468 17203473

[70] Krasemann S Madore C Cialic R Baufeld C Calcagno N El Fatimy R. The TREM2-APOE pathway drives the transcriptional phenotype of dysfunctional microglia in neurodegenerative diseases. Immunity. (2017) 47:566–81.e9. 10.1016/j.immuni.2017.08.008 28930663 PMC5719893

[71] Haynes SE Hollopeter G Yang G Kurpius D Dailey ME Gan W-B. The P2Y12 receptor regulates microglial activation by extracellular nucleotides. Nat Neurosci. (2006) 9:1512–9. 10.1038/nn1805 17115040

[72] Masuda T Amann L Sankowski R Staszewski O Lenz M D Errico P. Author correction: novel Hexb-based tools for studying microglia in the CNS. Nat Immunol. (2020) 21:1302. 10.1038/s41590-020-0774-6 32782404

[73] Grabert K McColl BW. Isolation and phenotyping of adult mouse microglial cells. Methods Mol Biol. (2018) 1784:77–86. 10.1007/978-1-4939-7837-3_7 29761389

[74] Barres BA. Designing and troubleshooting immunopanning protocols for purifying neural cells. Cold Spring Harb Protoc. (2014) 2014:1342–47. 10.1101/pdb.ip073999 25447277

[75] Bohlen CJ Friedman BA Dejanovic B Sheng M. Microglia in brain development, homeostasis, and neurodegeneration. Annu Rev Genet. (2019) 53:263–88. 10.1146/annurev-genet-112618-043515 31518519

[76] Tsatas O Ghasemlou N. Isolation and RNA purification of macrophages/microglia from the adult mouse spinal cord. J Immunol Methods. (2020) 477:112678. 10.1016/j.jim.2019.112678 31626757

[77] Moussaud S Draheim HJ. A new method to isolate microglia from adult mice and culture them for an extended period of time. J Neurosci Methods. (2010) 187:243–53. 10.1016/j.jneumeth.2010.01.017 20097228

[78] Butovsky O Jedrychowski MP Cialic R Krasemann S Murugaiyan G Fanek Z. Targeting miR-155 restores abnormal microglia and attenuates disease in SOD1 mice. Ann Neurol. (2015) 77:75–99. 10.1002/ana.24304 25381879 PMC4432483

[79] Gosselin D Skola D Coufal NG Holtman IR Schlachetzki JCM Sajti E. An environment-dependent transcriptional network specifies human microglia identity. Science. (2017) 356:eaal3222–13. 10.1126/science.aal3222 28546318 PMC5858585

[80] Galatro TF Holtman IR Lerario AM Vainchtein ID Brouwer N Sola PR. Transcriptomic analysis of purified human cortical microglia reveals age-associated changes. Nat Neurosci. (2017) 20:1162–71. 10.1038/nn.4597 28671693

[81] Smith AM Dragunow M. The human side of microglia. Trends Neurosci. (2014) 37:125–35. 10.1016/j.tins.2013.12.001 24388427

[82] Abud EM Ramirez RN Martinez ES Healy LM Nguyen CHH Newman SA. iPSC-derived human microglia-like cells to study neurological diseases. Neuron. (2017) 94:278–93.e9. 10.1016/j.neuron.2017.03.042 28426964 PMC5482419

[83] Douvaras P Sun B Wang M Kruglikov I Lallos G Zimmer M. Directed differentiation of human pluripotent stem cells to microglia. Stem Cell Rep. (2017) 8:1516–24. 10.1016/j.stemcr.2017.04.023 28528700 PMC5470097

[84] Haenseler W Sansom SN Buchrieser J Newey SE Moore CS Nicholls FJ. A highly efficient human pluripotent stem cell microglia model displays a neuronal-co-culture-specific expression profile and inflammatory response. Stem Cell Rep. (2017) 8:1727–42. 10.1016/j.stemcr.2017.05.017 28591653 PMC5470330

[85] Muffat J Li Y Yuan B Mitalipova M Omer A Corcoran S. Efficient derivation of microglia-like cells from human pluripotent stem cells. Nat Med. (2016) 22:1358–67. 10.1038/nm.4189 27668937 PMC5101156

[86] Pandya H Shen MJ Ichikawa DM Sedlock AB Choi Y Johnson KR. Differentiation of human and murine induced pluripotent stem cells to microglia-like cells. Nat Neurosci. (2017) 20:753–9. 10.1038/nn.4534 28253233 PMC5404968

[87] Takata K Kozaki T Lee CZW Thion MS Otsuka M Lim S. Induced-pluripotent-stem-cell-derived primitive macrophages provide a platform for modeling tissue-resident macrophage differentiation and function. Immunity. (2017) 47:183–98.e6. 10.1016/j.immuni.2017.06.017 28723550

[88] Kierdorf K Erny D Goldmann T Sander V Schulz C Perdiguero EG. Microglia emerge from erythromyeloid precursors via Pu.1- and Irf8-dependent pathways. Nat Neurosci. (2013) 16:273–80. 10.1038/nn.3318 23334579

[89] Schulz C Gomez Perdiguero E Chorro L Szabo-Rogers H Cagnard N Kierdorf K. A lineage of myeloid cells independent of Myb and hematopoietic stem cells. Science. (2012) 336:86–90. 10.1126/science.1219179 22442384

[90] Pocock JM Piers TM. Modelling microglial function with induced pluripotent stem cells: an update. Nat Rev Neurosci. (2018) 19:445–52. 10.1038/s41583-018-0030-3 29977068

[91] Hasselmann J Blurton-Jones M. Human iPSC-derived microglia: a growing toolset to study the brain's innate immune cells. Glia. (2020) 68:721–39. 10.1002/glia.23781 31926038 PMC7813153

[92] Gingras M Gagnon V Minotti S Durham HD Berthod F. Optimized protocols for isolation of primary motor neurons, astrocytes and microglia from embryonic mouse spinal cord. J Neurosci Methods. (2007) 163:111–8. 10.1016/j.jneumeth.2007.02.024 17445905

[93] Goshi N Morgan RK Lein PJ Seker E. A primary neural cell culture model to study neuron, astrocyte, and microglia interactions in neuroinflammation. J Neuroinflamm. (2020) 17:155. 10.1186/s12974-020-01819-z 32393376 PMC7216677

[94] Saura J Tusell JM Serratosa J. High-yield isolation of murine microglia by mild trypsinization. Glia. (2003) 44:183–89. 10.1002/glia.10274 14603460

[95] Lin L Desai R Wang X Lo EH Xing C. Characteristics of primary rat microglia isolated from mixed cultures using two different methods. J Neuroinflamm. (2017) 14:1–0. 10.1186/s12974-017-0877-7 28482909 PMC5422983

[96] He Y Yao X Taylor N Bai Y Lovenberg T Bhattacharya A. RNA sequencing analysis reveals quiescent microglia isolation methods from postnatal mouse brains and limitations of BV2 cells. J Neuroinflamm. (2018) 15:153. 10.1186/s12974-018-1195-4 29788964 PMC5964710

[97] Lawson LJ Perry VH Dri P Gordon S. Heterogeneity in the distribution and morphology of microglia in the normal adult mouse brain. Neuroscience. (1990) 39:151–70. 10.1016/0306-4522(90)90229-W 2089275

[98] Cuadros MA Martin C Coltey P Almendros A Navascués J. First appearance, distribution, and origin of macrophages in the early development of the avian central nervous system. J Comp Neurol. (1993) 330:113–29. 10.1002/cne.903300110 8468399

[99] Pan J Ma N Yu B Zhang W Wan J. Transcriptomic profiling of microglia and astrocytes throughout aging. J Neuroinflamm. (2020) 17:97. 10.1186/s12974-020-01774-9 32238175 PMC7115095

[100] Allendorf DH Puigdellívol M Brown GC. Activated microglia desialylate their surface, stimulating complement receptor 3-mediated phagocytosis of neurons. Glia. (2020) 68:989–98. 10.1002/glia.23757 31774586 PMC7079032

[101] Lecca D Janda E Mulas G Diana A Martino C Angius F. Boosting phagocytosis and anti-inflammatory phenotype in microglia mediates neuroprotection by PPARγ agonist MDG548 in Parkinson's disease models. Br J Pharmacol. (2018) 175:3298–314. 10.1111/bph.14214 29570770 PMC6057897

[102] Keaney J Gasser J Gillet G Scholz D Kadiu I. Inhibition of Bruton's tyrosine kinase modulates microglial phagocytosis: therapeutic implications for Alzheimer's disease. J Neuroimmune Pharmacol. (2019) 14:448–61. 10.1007/s11481-019-09839-0 30758770 PMC6707957

[103] Sellgren CM Gracias J Watmuff B Biag JD Thanos JM Whittredge PB. Increased synapse elimination by microglia in schizophrenia patient-derived models of synaptic pruning. Nat Neurosci. (2019) 22:374–85. 10.1038/s41593-018-0334-7 30718903 PMC6410571

[104] Sellgren CM Sheridan SD Gracias J Xuan D Fu T Perlis RH. Patient-specific models of microglia-mediated engulfment of synapses and neural progenitors. Mol Psychiatry. (2017) 22:170–7. 10.1038/mp.2016.220 27956744 PMC5285468

[105] Zabel MK Zhao L Zhang Y Gonzalez SR Ma W Wang X. Microglial phagocytosis and activation underlying photoreceptor degeneration is regulated by CX3CL1-CX3CR1 signaling in a mouse model of retinitis pigmentosa. Glia. (2016) 64:1479–91. 10.1002/glia.23016 27314452 PMC4958518

[106] Schulz D Severin Y Zanotelli VRT Bodenmiller B. In-depth characterization of monocyte-derived macrophages using a mass cytometry-based phagocytosis assay. Sci Rep. (2019) 9:1925. 10.1038/s41598-018-38127-9 30760760 PMC6374473

[107] Korn ED Weisman RA. Phagocytosis of latex beads by *Acanthamoeba*. II. Electron microscopic study of the initial events. J Cell Biol. (1967) 34:219–27. 10.1083/jcb.34.1.219 6033533 PMC2107219

[108] Desjardins M Huber LA Parton RG Griffiths G. Biogenesis of phagolysosomes proceeds through a sequential series of interactions with the endocytic apparatus. J Cell Biol. (1994) 124:677–88. 10.1083/jcb.124.5.677 8120091 PMC2119957

[109] Desjardins M Griffiths G. Phagocytosis: latex leads the way. Curr Opin Cell Biol. (2003) 15:498–503. 10.1016/S0955-0674(03)00083-8 12892792

[110] Pul R Chittappen KP Stangel M. Quantification of microglial phagocytosis by a flow cytometer-based assay. Methods Mol Biol. (2013) 1041:121–7. 10.1007/978-1-62703-520-0_14 23813376

[111] Yin Y Qiu S Li X Huang B Xu Y Peng Y. EZH2 suppression in glioblastoma shifts microglia toward M1 phenotype in tumor microenvironment. J Neuroinflamm. (2017) 14:1–1. 10.1186/s12974-017-0993-4 29132376 PMC5684749

[112] Dunn PA Eaton WR Lopatin ED McEntire JE Papermaster BW. Lymphokine-stimulated macrophage phagocytosis of fluorescent microspheres: a rapid new assay. J Immunol Methods. (1983) 64:71–83. 10.1016/0022-1759(83)90385-X 6580345

[113] Burleson GR Fuller LB Ménache MG Graham JA. Poly(I):poly(C)-enhanced alveolar and peritoneal macrophage phagocytosis: quantification by a new method utilizing fluorescent beads. Proc Soc Exp Biol Med Soc Exp Biol Med. (1987) 184:468–76. 10.3181/00379727-184-42501 3562455

[114] Hughes MM Field RH Perry VH Murray CL Cunningham C. Microglia in the degenerating brain are capable of phagocytosis of beads and of apoptotic cells, but do not efficiently remove PrPSc, even upon LPS stimulation. Glia. (2010) 58:2017–30. 10.1002/glia.21070 20878768 PMC3498730

[115] Kobayashi Y Inagawa H Kohchi C Okazaki K Zhang R Kobara H. Lipopolysaccharides derived from pantoea agglomerans can promote the phagocytic activity of amyloid in mouse microglial cells. Anticancer Res. (2017) 37:3917–20. 10.21873/anticanres.11774 28668895

[116] Ryu K-Y Cho G-S Piao HZ Kim W-K. Role of TGF-β in survival of phagocytizing microglia: autocrine suppression of TNF-α production and oxidative stress. Exp Neurobiol. (2012) 21:151–7. 10.5607/en.2012.21.4.151 23319875 PMC3538179

[117] Crespo-Castrillo A Garcia-Segura L-M Arevalo M-A. The synthetic steroid tibolone exerts sex-specific regulation of astrocyte phagocytosis under basal conditions and after an inflammatory challenge. J Neuroinflamm. (2020) 17:37. 10.1186/s12974-020-1719-6 31992325 PMC6986022

[118] Huang Y Mucke L. Alzheimer mechanisms and therapeutic strategies. Cell. (2012) 148:1204–22. 10.1016/j.cell.2012.02.040 22424230 PMC3319071

[119] Tanzi RE. A brief history of Alzheimer's disease gene discovery. J Alzheimers Dis. (2013) 33(Suppl. 1):S5–13. 10.3233/JAD-2012-129044 22986781

[120] Deczkowska A Keren-Shaul H Weiner A Colonna M Schwartz M Amit I. Disease-associated microglia: a universal immune sensor of neurodegeneration. Cell. (2018) 173:1073–81. 10.1016/j.cell.2018.05.003 29775591

[121] Nagano T Kimura SH Takemura M. Prostaglandin E2 reduces amyloid β-induced phagocytosis in cultured rat microglia. Brain Res. (2010) 1323:11–7. 10.1016/j.brainres.2010.01.086 20144888

[122] Hambardzumyan D Gutmann DH Kettenmann H. The role of microglia and macrophages in glioma maintenance and progression. Nat Neurosci. (2016) 19:20–7. 10.1038/nn.4185 26713745 PMC4876023

[123] Witkowska A Jahn R. Rapid SNARE-mediated fusion of liposomes and chromaffin granules with giant unilamellar vesicles. Biophys J. (2017) 113:1251–9. 10.1016/j.bpj.2017.03.010 28400045 PMC5607038

[124] Rideau E Dimova R Schwille P Wurm FR Landfester K. Liposomes and polymersomes: a comparative review towards cell mimicking. Chem Soc Rev. (2018) 47:8572–610. 10.1039/C8CS00162F 30177983

[125] Witkowska A Jablonski L Jahn R. A convenient protocol for generating giant unilamellar vesicles containing SNARE proteins using electroformation. Sci Rep. (2018) 8:1–8. 10.1038/s41598-018-27456-4 29930377 PMC6013450

[126] Roerdink F Wassef NM Richardson EC Alving CR. Effects of negatively charged lipids on phagocytosis of liposomes opsonized by complement. Biochim Biophys Acta. (1983) 734:33–9. 10.1016/0005-2736(83)90071-8 6615828

[127] Wang Y Cella M Mallinson K Ulrich JD Young KL Robinette ML. TREM2 lipid sensing sustains the microglial response in an Alzheimer's disease model. Cell. (2015) 160:1061–71. 10.1016/j.cell.2015.01.049 25728668 PMC4477963

[128] Urbaneja MA Goñi FM Alonso A. Structural changes induced by Triton X-100 on sonicated phosphatidylcholine liposomes. Eur J Biochem. (1988) 173:585–8. 10.1111/j.1432-1033.1988.tb14039.x 3371349

[129] Bangham AD. Physical structure and behavior of lipids and lipid enzymes. Adv Lipid Res. (1963) 1:65–104. 10.1016/B978-1-4831-9937-5.50008-9 14248958

[130] Hashioka S Han Y-H Fujii S Kato T Monji A Utsumi H. Phosphatidylserine and phosphatidylcholine-containing liposomes inhibit amyloid beta and interferon-gamma-induced microglial activation. Free Radic Biol Med. (2007) 42:945–54. 10.1016/j.freeradbiomed.2006.12.003 17349923

[131] Wu Z Nakanishi H. Phosphatidylserine-containing liposomes: potential pharmacological interventions against inflammatory and immune diseases through the production of prostaglandin E(2) after uptake by myeloid derived phagocytes. Arch Immunol Ther Exp. (2011) 59:195–201. 10.1007/s00005-011-0123-4 21479802

[132] Takayama F Wu Z Ma HM Okada R Hayashi Y Nakanishi H. Possible involvement of aiPLA2 in the phosphatidylserine-containing liposomes induced production of PGE2 and PGD2 in microglia. J Neuroimmunol. (2013) 262:121–4. 10.1016/j.jneuroim.2013.06.011 23850486

[133] Sierra A Abiega O Shahraz A Neumann H. Janus-faced microglia: beneficial and detrimental consequences of microglial phagocytosis. Front Cell Neurosci. (2013) 7:6. 10.3389/fncel.2013.00006 23386811 PMC3558702

[134] Mattson MP Keller JN Begley JG. Evidence for synaptic apoptosis. Exp Neurol. (1998) 153:35–48. 10.1006/exnr.1998.6863 9743565

[135] Nonaka S Nakanishi H. Microglial clearance of focal apoptotic synapses. Neurosci Lett. (2019) 707:134317. 10.1016/j.neulet.2019.134317 31175934

[136] Witting A Müller P Herrmann A Kettenmann H Nolte C. Phagocytic clearance of apoptotic neurons by microglia/brain macrophages *in vitro*: involvement of lectin-, integrin-, and phosphatidylserine-mediated recognition. J Neurochem. (2000) 75:1060–70. 10.1046/j.1471-4159.2000.0751060.x 10936187

[137] Zhao X Zhang L Ting S-M Aronowski J. Phagocytosis assay of microglia for dead neurons in primary rat brain cell cultures. Bio-protocol. (2016) 6:e1795. 10.21769/BioProtoc.1795 29552587 PMC5856257

[138] Zhao X Wang H Sun G Zhang J Edwards NJ Aronowski J. Neuronal interleukin-4 as a modulator of microglial pathways and ischemic brain damage. J Neurosci. (2015) 35:11281–91. 10.1523/JNEUROSCI.1685-15.2015 26269636 PMC4532758

[139] McLaughlin CN Perry-Richardson JJ Coutinho-Budd JC Broihier HT. Dying neurons utilize innate immune signaling to prime glia for phagocytosis during development. Dev Cell. (2019) 48:506–22.e6. 10.1016/j.devcel.2018.12.019 30745142 PMC6394877

[140] Takahashi K Rochford CDPP Neumann H. Clearance of apoptotic neurons without inflammation by microglial triggering receptor expressed on myeloid cells-2. J Exp Med. (2005) 201:647–57. 10.1084/jem.20041611 15728241 PMC2213053

[141] Neumann H Takahashi K. Essential role of the microglial triggering receptor expressed on myeloid cells-2 (TREM2) for central nervous tissue immune homeostasis. J Neuroimmunol. (2007) 184:92–9. 10.1016/j.jneuroim.2006.11.032 17239445

[142] Beccari S Diaz-Aparicio I Sierra A. Quantifying microglial phagocytosis of apoptotic cells in the brain in health and disease. Curr Protoc Immunol. (2018) 122:e49. 10.1002/cpim.49 29927067

[143] Diaz-Aparicio I Paris I Sierra-Torre V Plaza-Zabala A Rodríguez-Iglesias N Márquez-Ropero M. Microglia actively remodel adult hippocampal neurogenesis through the phagocytosis secretome. J Neurosci. (2020) 40:1453–82. 10.1523/JNEUROSCI.0993-19.2019 31896673 PMC7044727

[144] Ahmad F Liu P. Synaptosome as a tool in Alzheimer's disease research. Brain Res. (2020) 1746:147009. 10.1016/j.brainres.2020.147009 32659233

[145] Evans GJO. The synaptosome as a model system for studying synaptic physiology. Cold Spring Harb Protoc. (2015) 2015:421–4. 10.1101/pdb.top074450 25934942

[146] Nicholls DG Sihra TS. Synaptosomes possess an exocytotic pool of glutamate. Nature. (1986) 321:772–3. 10.1038/321772a0 3713864

[147] Byun YG Chung WS. A novel *in vitro* live-imaging assay of astrocyte-mediated phagocytosis using pH indicator-conjugated synaptosomes. J Vis Exp. (2018) 2018:56647. 10.3791/56647 29443098 PMC5912350

[148] Madore C Leyrolle Q Morel L Rossitto M Greenhalgh AD Delpech JC. Essential omega-3 fatty acids tune microglial phagocytosis of synaptic elements in the mouse developing brain. Nat Commun. (2020) 11:6133. 10.1038/s41467-020-19861-z 33257673 PMC7704669

[149] Evans AK Ardestani PM Yi B Park HH Lam RK Shamloo M. Beta-adrenergic receptor antagonism is proinflammatory and exacerbates neuroinflammation in a mouse model of Alzheimer's Disease. Neurobiol Dis. (2020) 146:105089. 10.1016/j.nbd.2020.105089 32971233 PMC7686098

[150] Chung WS Clarke LE Wang GX Stafford BK Sher A Chakraborty C. Astrocytes mediate synapse elimination through MEGF10 and MERTK pathways. Nature. (2013) 504:394–400. 10.1038/nature12776 24270812 PMC3969024

[151] Ivannikov MV Sugimori M Llinás RR. Synaptic vesicle exocytosis in hippocampal synaptosomes correlates directly with total mitochondrial volume. J Mol Neurosci. (2013) 49:223–30. 10.1007/s12031-012-9848-8 22772899 PMC3488359

[152] Kim H-J Cho M-H Shim WH Kim JK Jeon E-Y Kim D-H. Deficient autophagy in microglia impairs synaptic pruning and causes social behavioral defects. Mol Psychiatry. (2017) 22:1576–84. 10.1038/mp.2016.103 27400854 PMC5658669

[153] Jahn A Vreeland WN DeVoe DL Locascio LE Gaitan M. Microfluidic directed formation of liposomes of controlled size. Langmuir. (2007) 23:6289–93. 10.1021/la070051a 17451256

[154] Lim JP Gleeson PA. Macropinocytosis: an endocytic pathway for internalising large gulps. Immunol Cell Biol. (2011) 89:836–43. 10.1038/icb.2011.20 21423264

[155] Lim JP Gosavi P Mintern JD Ross EM Gleeson PA. Sorting nexin 5 selectively regulates dorsal-ruffle-mediated macropinocytosis in primary macrophages. J Cell Sci. (2015) 128:4407–19. 10.1242/jcs.174359 26459636

[156] Gylys KH Fein JA Wiley DJ Cole GM. Rapid annexin-V labeling in synaptosomes. Neurochem Int. (2004) 44:125–31. 10.1016/S0197-0186(03)00146-3 14568554

[157] Morsch M Radford R Lee A Don EK Badrock AP Hall TE. *In vivo* characterization of microglial engulfment of dying neurons in the zebrafish spinal cord. Front Cell Neurosci. (2015) 9:321. 10.3389/fncel.2015.00321 26379496 PMC4553390

[158] Damisah EC Hill RA Rai A Chen F Rothlin CV Ghosh S. Astrocytes and microglia play orchestrated roles and respect phagocytic territories during neuronal corpse removal *in vivo*. Sci Adv. (2020) 6:eaba3239. 10.1126/sciadv.aba3239 32637606 PMC7319765

[159] Tejera D Heneka MT. *In vivo* phagocytosis analysis of amyloid beta. Methods Mol Biol. (2019) 2034:287–92. 10.1007/978-1-4939-9658-2_21 31392693

[160] Brioschi S Zhou Y Colonna M. Brain parenchymal and extraparenchymal macrophages in development, homeostasis, and disease. J Immunol. (2020) 204:294–305. 10.4049/jimmunol.1900821 31907272 PMC7034672

[161] Roqué PJ Costa LG. Co-culture of neurons and microglia. Curr Protoc Toxicol. (2017) 74:11.24.1–17. 10.1002/cptx.32 29117434 PMC5774987

[162] Harry GJ Kraft AD. Neuroinflammation and microglia: considerations and approaches for neurotoxicity assessment. Expert Opin Drug Metab Toxicol. (2008) 4:1265–277. 10.1517/17425255.4.10.1265 18798697 PMC2658618

[163] Neher JJ Neniskyte U Zhao J-W Bal-Price A Tolkovsky AM Brown GC. Inhibition of microglial phagocytosis is sufficient to prevent inflammatory neuronal death. J Immunol. (2011) 186:4973–83. 10.4049/jimmunol.1003600 21402900

[164] Fricker M Oliva-Martín MJ Brown GC. Primary phagocytosis of viable neurons by microglia activated with LPS or Aβ is dependent on calreticulin/LRP phagocytic signalling. J Neuroinflamm. (2012) 9:196. 10.1186/1742-2094-9-196 PMC3481398 22889139

[165] Manelli AM Bulfinch LC Sullivan PM LaDu MJ. Abeta42 neurotoxicity in primary co-cultures: effect of apoE isoform and Abeta conformation. Neurobiol Aging. (2007) 28:1139–147. 10.1016/j.neurobiolaging.2006.05.024 16837105 PMC3752940

[166] Lui H Zhang J Makinson SR Cahill MK Kelley KW Huang H-Y. Progranulin deficiency promotes circuit-specific synaptic pruning by microglia via complement activation. Cell. (2016) 165:921–35. 10.1016/j.cell.2016.04.001 27114033 PMC4860138

[167] Kao AW Eisenhut RJ Martens LH Nakamura A Huang A Bagley JA. A neurodegenerative disease mutation that accelerates the clearance of apoptotic cells. Proc Natl Acad Sci USA. (2011) 108:4441–6. 10.1073/pnas.1100650108 21368173 PMC3060230

[168] Martens LH Zhang J Barmada SJ Zhou P Kamiya S Sun B. Progranulin deficiency promotes neuroinflammation and neuron loss following toxin-induced injury. J Clin Invest. (2012) 122:3955–9. 10.1172/JCI63113 23041626 PMC3484443

[169] Sarn N Jaini R Thacker S Lee H Dutta R Eng C. Cytoplasmic-predominant Pten increases microglial activation and synaptic pruning in a murine model with autism-like phenotype. Mol Psychiatry. (2020). 10.1038/s41380-020-0681-0. [Epub ahead of print]. 32055008 PMC8159731

[170] Ito D Imai Y Ohsawa K Nakajima K Fukuuchi Y Kohsaka S. Microglia-specific localisation of a novel calcium binding protein, Iba1. Mol Brain Res. (1998) 57:1–9. 10.1016/S0169-328X(98)00040-0 9630473

[171] Lee JE Liang KJ Fariss RN Wong WT. *Ex vivo* dynamic imaging of retinal microglia using time-lapse confocal microscopy. Invest Ophthalmol Vis Sci. (2008) 49:4169–76. 10.1167/iovs.08-2076 18487378 PMC2652634

[172] Dailey ME Eyo U Fuller L Hass J Kurpius D. Imaging microglia in brain slices and slice cultures. Cold Spring Harb Protoc. (2013) 2013:1142–8. 10.1101/pdb.prot079483 24298036

[173] Nimmerjahn A Kirchhoff F Helmchen F. Neuroscience: resting microglial cells are highly dynamic surveillants of brain parenchyma *in vivo*. Science. (2005) 308:1314–8. 10.1126/science.1110647 15831717

[174] Stence N Waite M Dailey ME. Dynamics of microglial activation: a confocal time-lapse analysis in hippocampal slices. Glia. (2001) 33:256–66. 10.1002/1098-1136(200103)33:3<256::AID-GLIA1024>3.0.CO;2-J 11241743

[175] Bisht K Sharma KP Lecours C Gabriela Sánchez M El Hajj H Milior G. Dark microglia: a new phenotype predominantly associated with pathological states. Glia. (2016) 64:826–39. 10.1002/glia.22966 26847266 PMC4949554

[176] Sipe GO Lowery RL Tremblay M Kelly EA Lamantia CE Majewska AK. Microglial P2Y12 is necessary for synaptic plasticity in mouse visual cortex. Nat Commun. (2016) 7:10905. 10.1038/ncomms10905 26948129 PMC4786684

[177] Villani A Benjaminsen J Moritz C Henke K Hartmann J Norlin N. Clearance by microglia depends on packaging of phagosomes into a unique cellular compartment. Dev Cell. (2019) 49:77–88.e7. 10.1016/j.devcel.2019.02.014 30880002

[178] Berg S Kutra D Kroeger T Straehle CN Kausler BX Haubold C. ilastik: interactive machine learning for (bio)image analysis. Nat Methods. (2019) 16:1226–32. 10.1038/s41592-019-0582-9 31570887

[179] McQuin C Goodman A Chernyshev V Kamentsky L Cimini BA Karhohs KW. CellProfiler 3.0: next-generation image processing for biology. PLoS Biol. (2018) 16:1–7. 10.1371/journal.pbio.2005970 29969450 PMC6029841

[180] Lauro C Catalano M Trettel F Limatola C. Fractalkine in the nervous system: neuroprotective or neurotoxic molecule? Ann N Y Acad Sci. (2015) 1351:141–8. 10.1111/nyas.12805 26084002

[181] Pawelec P Ziemka-Nalecz M Sypecka J Zalewska T. The impact of the CX3CL1/CX3CR1 axis in neurological disorders. Cells. (2020) 9:2277. 10.3390/cells9102277 33065974 PMC7600611

[182] Schafer DP Lehrman EK Heller CT Stevens B. An engulfment assay: a protocol to assess interactions between CNS phagocytes and neurons. J Vis Exp. (2014) 88:51482. 10.3791/51482 24962472 PMC4188069

[183] Cignarella F Filipello F Bollman B Cantoni C Locca A Mikesell R. TREM2 activation on microglia promotes myelin debris clearance and remyelination in a model of multiple sclerosis. Acta Neuropathol. (2020) 140:513–34. 10.1007/s00401-020-02193-z 32772264 PMC7498497

[184] Rangaraju S Raza SA Li NX Betarbet R Dammer EB Duong D. Differential phagocytic properties of CD45(low) microglia and CD45(high) brain mononuclear phagocytes-activation and age-related effects. Front Immunol. (2018) 9:405. 10.3389/fimmu.2018.00405 29552013 PMC5840283

[185] Bandura DR Baranov VI Ornatsky OI Antonov A Kinach R Lou X. Mass cytometry: technique for real time single cell multitarget immunoassay based on inductively coupled plasma time-of-flight mass spectrometry. Anal Chem. (2009) 81:6813–22. 10.1021/ac901049w 19601617

[186] Ornatsky O Bandura D Baranov V Nitz M Winnik MA Tanner S. Highly multiparametric analysis by mass cytometry. J Immunol Methods. (2010) 361:1–20. 10.1016/j.jim.2010.07.002 20655312

[187] Bendall SC Simonds EF Qiu P Amir ED Krutzik PO Finck R. Single-cell mass cytometry of differential immune and drug responses across a human hematopoietic continuum. Science. (2011) 332:687–96. 10.1126/science.1198704 21551058 PMC3273988

[188] Guilliams M Dutertre C-A Scott CL McGovern N Sichien D Chakarov S. Unsupervised high-dimensional analysis aligns dendritic cells across tissues and species. Immunity. (2016) 45:669–84. 10.1016/j.immuni.2016.08.015 27637149 PMC5040826

[189] Becher B Schlitzer A Chen J Mair F Sumatoh HR Teng KWW. High-dimensional analysis of the murine myeloid cell system. Nat Immunol. (2014) 15:1181–9. 10.1038/ni.3006 25306126

[190] Ueda HR Ertürk A Chung K Gradinaru V Chédotal A Tomancak P. Publisher correction: tissue clearing and its applications in neuroscience. Nat Rev Neurosci. (2020) 21:298. 10.1038/s41583-020-0291-5 32152524

[191] Helmchen F Denk W. Deep tissue two-photon microscopy. Nat Methods. (2005) 2:932–40. 10.1038/nmeth818 16299478

[192] Attardo A Fitzgerald JE Schnitzer MJ. Impermanence of dendritic spines in live adult CA1 hippocampus. Nature. (2015) 523:592–6. 10.1038/nature14467 26098371 PMC4648621

